# KIFC1 is activated by TCF-4 and promotes hepatocellular carcinoma pathogenesis by regulating HMGA1 transcriptional activity

**DOI:** 10.1186/s13046-019-1331-8

**Published:** 2019-07-24

**Authors:** Kai Teng, Shi Wei, Chi Zhang, Jiewei Chen, Jinbin Chen, Kanghua Xiao, Jun Liu, Miaomiao Dai, Xinyuan Guan, Jingping Yun, Dan Xie

**Affiliations:** 1 State Key Laboratory of Oncology in South China, Collaborative Innovation Center for Cancer Medicine, Sun Yat-sen University Cancer Center, Guangzhou, 510060 China; 2 Department of Pathology, Sun Yat-sen University Cancer Center, Guangzhou, 510060 China; 3 Department of Hepatobiliary Oncology, Sun Yat-sen University Cancer Center, Guangzhou, 510060 China; 4 Department of Urology, Sun Yat-sen University Cancer Center, Guangzhou, 510060 China

**Keywords:** Hepatocellular carcinoma, Kinesin family member C1, Pathogenesis, TCF-4, HMGA1

## Abstract

**Background:**

Kinesins play important roles in the development and progression of many human cancers. The functions and underlying mechanisms of kinesin family member C1 (KIFC1), a member of the kinesin-14 family, in the pathogenesis of hepatocellular carcinoma (HCC) have not been fully elucidated.

**Methods:**

In this study, 168 HCC samples were first analyzed to examine the association between KIFC1 expression and patient clinicopathological features and prognosis. The role of KIFC1 in HCC cell proliferation and metastasis was investigated both in vivo and in vitro. The upstream regulation and downstream targets of KIFC1 were studied by qRT-PCR, western blotting, coimmunoprecipitation, chromatin immunoprecipitation (ChIP) and dual-luciferase reporter assays.

**Results:**

KIFC1 was highly expressed in HCC tissues and positively associated with advanced stages and poor prognosis. KIFC1 knockdown suppressed HCC cell proliferation and invasion both in vitro and in vivo. Furthermore, KIFC1 knockdown decreased invadopodia formation and reduced epithelial-mesenchymal transition (EMT). HMGA1, an architectural transcriptional factor, was identified to interact with KIFC1. HMGA1 could bind to the promoters of Stat3, MMP2 and EMT-related genes and promote gene transcription. KIFC1 enhanced HMGA1 transcriptional activity and facilitated HCC proliferation and invasion. Moreover, KIFC1 was activated by TCF-4, and KIFC1 inhibition enhanced HCC cell sensitivity to paclitaxel.

**Conclusions:**

Our findings suggest that KIFC1, activated by TCF-4, functions as an oncogene and promotes HCC pathogenesis through regulating HMGA1 transcriptional activity and that KIFC1 is a potential therapeutic target to enhance the paclitaxel sensitivity of HCC.

**Electronic supplementary material:**

The online version of this article (10.1186/s13046-019-1331-8) contains supplementary material, which is available to authorized users.

## Background

Hepatocellular carcinoma (HCC) accounts for 85–90% of all primary liver cancers. Approximately 850,000 new cases are reported each year globally, making it the second leading cause of cancer-related death worldwide. China has approximately 50% of the total number of HCC cases and deaths [1]. HCC has an insidious onset and gradual progression without apparent symptoms and is difficult to diagnose at an early stage. Patients usually present with symptoms at advanced stages. For these patients, there are limited effective therapies. Sorafenib, a multikinase inhibitor for HCC first-line therapy, can only increase survival by approximately 3 months. There is great urgency for HCC researchers to find new biomarkers to diagnose HCC at an early stage and competent targets to improve the dismal survival of patients.

Kinesin was first isolated from squid giant axon and was identified as molecular motors in 1985 by Vale [2]. A total of 45 human kinesin superfamily proteins (KIFs) have been discovered in humans to date. They are divided into 14 subfamilies based on structural differences (from KIF1 to KIF14). KIFs are microtubule-dependent molecular motor proteins with adenosine triphosphatase activity. The KIFs are involved in several cellular events, such as mitosis, meiosis and the transport of macromolecules [3].

It is indicated that the aberrant expression of kinesins plays critical roles in the tumorigenesis and progression of various human cancers [4]. Overexpression of KIF2A was associated with a poor prognosis in ovarian cancer [5]. KIF26B promotes proliferation and metastasis by activating the VEGF pathway in gastric cancer [6]. KIFs is also implicated in cancer chemoresistance. Kinesins are intracellular motor proteins that interact with microtubules, which are also the target for taxanes. Overexpression of the kinesin KIFC3 is significantly correlated with resistance to both docetaxel and paclitaxel, but not to platinum-based chemotherapy [7].

Kinesin family member C1 (KIFC1) belongs to the C-type kinesin of the kinesin-14 family. Kinesins are a family of molecular motor proteins that play important roles in intracellular transport and cell division. Studies have demonstrated that KIFC1 is involved in the pathogenesis and development of a variety of neoplasms, including breast cancer [8], ovarian cancer [9], prostate cancer [10], non-small cell lung cancer [11] and esophageal squamous cell carcinoma [12]. Few studies focused on KIFC1 expression and HCC pathogenesis at the time we designed this study. KIFC1 is normally a nonessential kinesin motor but plays important roles in cancer cells with extra centrosomes. It clusters centrosomes in cancer cells containing multiple centrosomes to avoid multipolar cell division. Thus, cancer cells can survive and continue to progress [3]. KIFC1 is a rational tumor-selective therapeutic target and deserves further exploration.

In this study, we investigated the role of KIFC1 in HCC pathogenesis and discovered that KIFC1 was highly expressed in both HCC cell lines and clinical samples. HCC proliferation and metastasis were inhibited in KIFC1-knockdown cells both in vitro and in vivo. Further study demonstrated that HMGA1 was the binding partner of KIFC1 and that KIFC1 modulated HMGA1 transcriptional activity. Moreover, KIFC1 was regulated by TCF-4, one key transcriptional factor in the Wnt/β-catenin pathway. Finally, KIFC1 inhibition increased HCC cell sensitivity to paclitaxel. Our research revealed the interaction between KIFC1 and HMGA1 in HCC cells, uncovered the underlying molecular mechanisms and demonstrated the potential clinical use of KIFC1 inhibition in future HCC therapy.

## Materials and methods

### Patients and tissue characteristics

HCC tissues (168 cases) and corresponding adjacent paired noncancerous liver tissues (30 cases) from patients who underwent hepatectomy between January 2013 and November 2015 were obtained from the archives of the Department of Pathology, Sun Yat-sen University Cancer Center (SYSUCC, Guangzhou, China). The human tissue microarray (TMA) was constructed according to a method described previously [13]. For mRNA and protein, another 40 paired HCC and adjacent specimens were obtained from the Biological Specimen Bank of Sun Yat-sen University Cancer Center. Written informed consent was obtained from all patients before the study. All the samples used in this study were approved by the Committees for Ethical Review of Research Involving Human Subjects at the Sun Yat-Sen University Cancer Center.

### Cell culture

Eight human HCC cell lines (HepG2, Hep3B, QGY-7701, BEL-7402, SMMC-7721, Huh-7, CRL-8024, and LM6) and two immortalized normal liver cell line, MIHA and LO2, were selected and cultured in this study. All cells were maintained with Dulbecco’s modified Eagle’s medium (DMEM, Invitrogen, Carlsbad, CA, USA) containing 10% (v/v) fetal bovine serum (PAN-biotech, Aidenbach, Germany) and 1% penicillin-streptomycin. All cells were cultured at 37 °C in a humidified incubator with 5% CO_2_.

### Quantitative real-time PCR

Total RNA was extracted using TRIzol reagent (Invitrogen, Carlsbad, CA, USA) and then reverse transcribed using the PrimeScript RT-PCR Kit (Takara, Dalian, China) according to the manufacturer’s instructions. A SYBR Green PCR Kit (Bio-Rad, Hercules, CA, USA) was used to conduct qRT-PCR. Relative quantities (Δ cycle threshold (Ct) values) were obtained by normalizing to GAPDH. The comparative Ct (2^- ΔΔCt^) method was adopted to analyze the qRT-PCR data. The primer sequences used in this assay are attached to Additional file 1: Table S1.

### Immunohistochemical staining

Tissue samples were dewaxed in xylene and dehydrated in a graded series of ethanol, followed by antigen retrieval in Tris/EDTA (pH = 8.0), and incubation with goat serum and primary and secondary antibodies as previously described [14]. The primary antibody against KIFC1 (1:200; ab172620, Abcam, Cambridge, UK) was used for IHC staining. A negative control was achieved by replacing the primary antibody with normal rabbit IgG. The staining result for KIFC1 was evaluated by two pathologists in a mutually blinded manner. A semiquantitative scoring criterion was adopted by recording both staining intensity and positively stained areas [15]. The staining intensity was scored as 0 (negative), 1 (weak), 2 (moderate), and 3 (strong). Staining extent was scored as 1 (1–10%), 2 (10–40%), 3 (40–75%), and 4 (75–100%) according to the percentage of positively stained cells. The final staining score was calculated by multiplying the staining intensity score by the staining extent score. The KIFC1 mean score was 3, and consequently, the specimens were divided into two groups, low KIFC1 (0–3) and high KIFC1 (3–12).

### Immunofluorescence assay

HCC cells cultured in a glass dish were fixed with 4% paraformaldehyde and permeabilized with 0.2% Triton X-100. After blocking with 10% goat serum, the cells were incubated with primary antibodies at 4 °C overnight (KIFC1, 1:100, sc-100947, Santa Cruz Biotechnology, Santa Cruz, CA, USA; HMGA1, 1:100, ab129153, Abcam, Cambridge, UK). The cell dish was then incubated with fluorescent secondary antibodies (Proteintech, Wuhan, China) or rhodamine-phalloidin (Invitrogen, Carlsbad, CA, USA) for 1 h at room temperature. Nuclei were stained with DAPI (Beyotime, Shanghai, China). Lastly, cells were imaged with a laser scanning confocal microscope (Olympus FV1000, Tokyo, Japan).

### Construction of the recombinant lentiviral vector

The KIFC1 shRNA lentiviral expression vector was purchased from GeneCopoeia Company (Rockville, MD, USA). The Psi-LVRU6GP vector was used to construct KIFC1 shRNA and control plasmids. Cell lines 7701 and 7402, with relatively high levels of KIFC1 expression, were chosen as target cell lines. Puromycin was employed to select stable cells. The target sequences of KIFC1 for constructing lentiviral shRNAs are resented in Additional file 1: Table S1.

### Plasmid constructs and transfection

The coding sequences (CDSs) of human KIFC1 and HMGA1 were amplified and cloned into the pLVX-myc-mcs-3xflag lentivirus vector. The corresponding empty vector served as a control. The KIFC1 sequence spanning 1000 bp near the transcriptional start site (TSS) and its truncated and mutated variants were amplified and cloned into the pGL3 vector (Promega, Madison, WI, USA). HEK-293 T cells were transfected with these plasmids using Lipofectamine™ 2000 (Invitrogen, Carlsbad, CA, USA) along with the packaging and envelope plasmids, psPAX2 and pMD2.G, according to the manufacturer’s protocol (Addgene, Watertown, MA, USA). Virus particles were harvested 48 h after transfection. HCC cells were then infected and selected with puromycin. The target primer sequences are presented in Additional file 1: Table S1.

### Generation of KIFC1 knock out cells using CRISPR/Cas9 system

The CRISPR/Cas9 single gRNA (sgRNA) was designed and assembled as previously described [16]. Protospacer sequences of CRISPR/Cas9 against KFIC1 were designed by Guide Design Resources (https://zlab.bio/guide-design-resources). All specific target sequences were amplified and cloned into lentiCRISPR v2 (Addgene, Cambrige, MA, USA) and verified by Sanger sequencing. Cell pellets were collected and genomic DNA was extracted at 48 h after transfection of CRISPR/Cas9 into 293 T cells. T7 endonuclease I was used to compare cleavage efficiencies of different sgRNAs. sgRNA3 and sgRNA4 were validated to be the effective sgRNA sequences (sgRNA3-F: CACCGTGGGAAGGGGCCTTAATCAG; sgRNA3-R: AAACCTGATTAAGGCCCCTTCCCAC; sgRNA4-F: CACCGTAATTTTGGTCGTTGCACCC; sgRNA4-R: AAACGGGTGCAACGACCAAAATTAC). Transfer plasmid lentiCRISPR v2 with sgRNA3 or sgRNA4 was co-transfected with packaging plasmids psPAX2 and pMD2.G to product lentivirus. HepG2 was infected with this lentivirus and selected with puromycin. After selection, Colonies derived from a single cell were expanded with conditioned media. Western blot was further used for knock out validation.

### Western blotting

Whole cells or tissue samples were lysed with RIPA buffer (Thermo Scientific, Carlsbad, CA, USA) containing protease cocktail inhibitors (Roche, Mannheim, Germany). After denaturation by SDS (sodium dodecyl sulfate), the protein lysate was electrophoresed by SDS-PAGE and transferred to a 0.22 μm PVDF membrane (Roche, Penzberg, Germany). The membrane was then blocked with 5% milk and incubated with the indicated primary antibody overnight at 4 °C. After washing with TBST and secondary antibody incubation for 1 h at room temperature, the membrane was visualized with an ECL development system (Tanon, Shanghai, China). The antibodies used in the WB assay are shown in Additional file 1: Table S2.

### Cell proliferation and colony formation assays

HCC cells were seeded in 96-well plates (1000 cells/well). After cell attachment, CCK-8 (MCE, Shanghai, China) was administered to test the OD value continuously for 5 days as described in the manuscript. For the colony formation assay, 500 or 1000 cells were placed in a 6-well plate and maintained with 10% FBS-DMEM for 2 weeks. Colonies were fixed with methanol and stained with 0.1% crystal violet for 15 min. A clone including more than 50 cells was counted as positive.

### Transwell migration and invasion assays

Cell migration assays were performed with an 8 μm Transwell chamber (Falcon,Tewksbury MA, USA) and a 24-well culture plate. Matrigel (5%, Corning, Tewksbury MA, USA) was precoated in the Transwell chamber and incubated for 1 h at 37 °C before the Transwell invasion assay. The remaining procedures followed standard instructions. HCC cells (5 × 10^4^) were suspended in 300 μL serum-free DMEM and added to the upper chamber. The lower chamber was filled with 600 μL 10% FBS-DMEM. After incubation for 24 h–36 h, non-migrated cells in the upper chamber were removed with a cotton swab, while those on the lower side were fixed with methanol and stained with crystal violet. Invaded cells were calculated under a microscope. Both assays were performed in triplicate independently.

### HCC mouse model

Female BALB/c athymic nude mice (4 weeks old) were used in this study. For the xenograft model, HCC cells (2 × 10^6^) were suspended in 100 μL serum-free DMEM and subcutaneously injected into the flanks of nude mice. Tumor size was measured weekly, and all mice were sacrificed 1 month after injection. For orthotopic implantation mouse models, mice were anesthetized with isoflurane. The surgery was conducted on the left liver lobe along the left rib edge. HCC cells (2 × 10^6^) were suspended in 20 μL PBS containing 25% Matrigel (Corning, New York, USA) and injected into the left lobe. The injection site was gently pressed with a cotton swab to reduce bleeding and leakage of the cell suspension. Finally, the peritoneum and skin were closed with 4–0 absorbable sutures (Jinhuan Medical, Shanghai, China). For the intravenous metastasis model, HCC cells (2 × 10^6^) were injected into the tail vein of each mouse. Eight weeks after implantation and vein injection, mice in both models were euthanized and examined. Tumor size was calculated as follows: tumor volume = (L × W^2^) / 2, where L = long axis and W = short axis. Livers and lungs were harvested, fixed with formalin, sectioned serially and stained with hematoxylin and eosin (H&E) for standard histological examination. Animal studies were approved by the SYSUCC Institutional Animal Care and Usage Committee and performed in accordance with SYSUCC animal care guidelines.

### Coimmunoprecipitation

Cell lysates were harvested and precleared by incubation with protein A/G beads (Thermo Scientific, Carlsbad, CA, USA) to eliminate nonspecific binding. Then, cell lysates were subjected to immunoprecipitation with Flag-tag (Cell Signaling Technology, Danvers, MA, USA) or Myc-tag (MBL, Nagoya, Japan) antibodies followed by incubation with protein A/G beads. The precipitates were washed and boiled to isolate bound proteins. The proteins obtained were subsequently analyzed by western blot, silver staining and mass spectrometry (MS). Silver staining was performed with the Fast Silver Stain Kit (Beyotime, Shanghai, China), and MS was carried out by Wininnovate Bio, Shenzhen, China. The original data and comparison between HepG2-vector and HepG2-KIFC1 are listed in the Additional file 2.

### Chromatin immunoprecipitation (ChIP)

The ChIP assay was performed with an EZ-Magna ChIP Kit (Millipore, Darmstadt, Germany) according to the manufacturer’s instructions. Briefly, cell lysate was sonicated to shear DNA into 200–500 bp fragments, and a ChIP grade antibody against HMGA1 (Abcam, ab4078, 1:200, Cambridge, UK) was used for immunoprecipitation. The ChIP product was quantified using qRT-PCR with primers specific to the regulated gene promoters. ChIP primers are listed in Additional file 1: Table S1.

### Dual-luciferase reporter assay

The KIFC1 promoter sequence was cloned into the pGL3-Basic plasmid. The Super TOPflash luciferase reporter vector served as a positive control of Wnt/β-catenin pathway activation. Renilla activities were analyzed using a dual luciferase assay kit (Vazyme, Nanjing, China). HEK-293 T cells were transfected with pGL3-Basic or modified vectors with truncated or mutant promoter sequences. The mutant KIFC1 promoter sequences were 5′-GCTTTGAATC-3′. Twenty-four hours after transfection, three inhibitors of various components from the Wnt/β-catenin pathway were added to stimulate or inhibit the Wnt/β-catenin pathway, including the Wnt pathway activator LiCl (a GSK3β inhibitor) and the Wnt pathway suppressor XAV939 (inhibiting Tankyrase, thereby stabilizing Axin2) or iCRT3 (an inhibitor disrupting the β-catenin-TCF-4 interaction). Reporter luciferase activity was normalized to Renilla luciferase activity (internal control), and all reporter gene assays were conducted in triplicate.

### Statistical analysis

Statistical analysis was performed using SPSS 20.0 software (IBM, Chicago, IL, USA). Survival curves were plotted by Kaplan–Meier analysis and compared with log-rank tests. Multivariate survival analysis was performed for all parameters found to be significant in the univariate analysis by using the Cox regression model. Bivariate correlations between studied variables were calculated by Pearson’s correlation coefficients. Comparisons between two groups for statistical significance were performed with an independent Student’s t test. Data are presented as the mean ± SD, and *P* values less than 0.05 were considered statistically significant.

## Results

### KIFC1 is highly expressed in HCC tissue and cancer cell lines

**Fig. 1:**
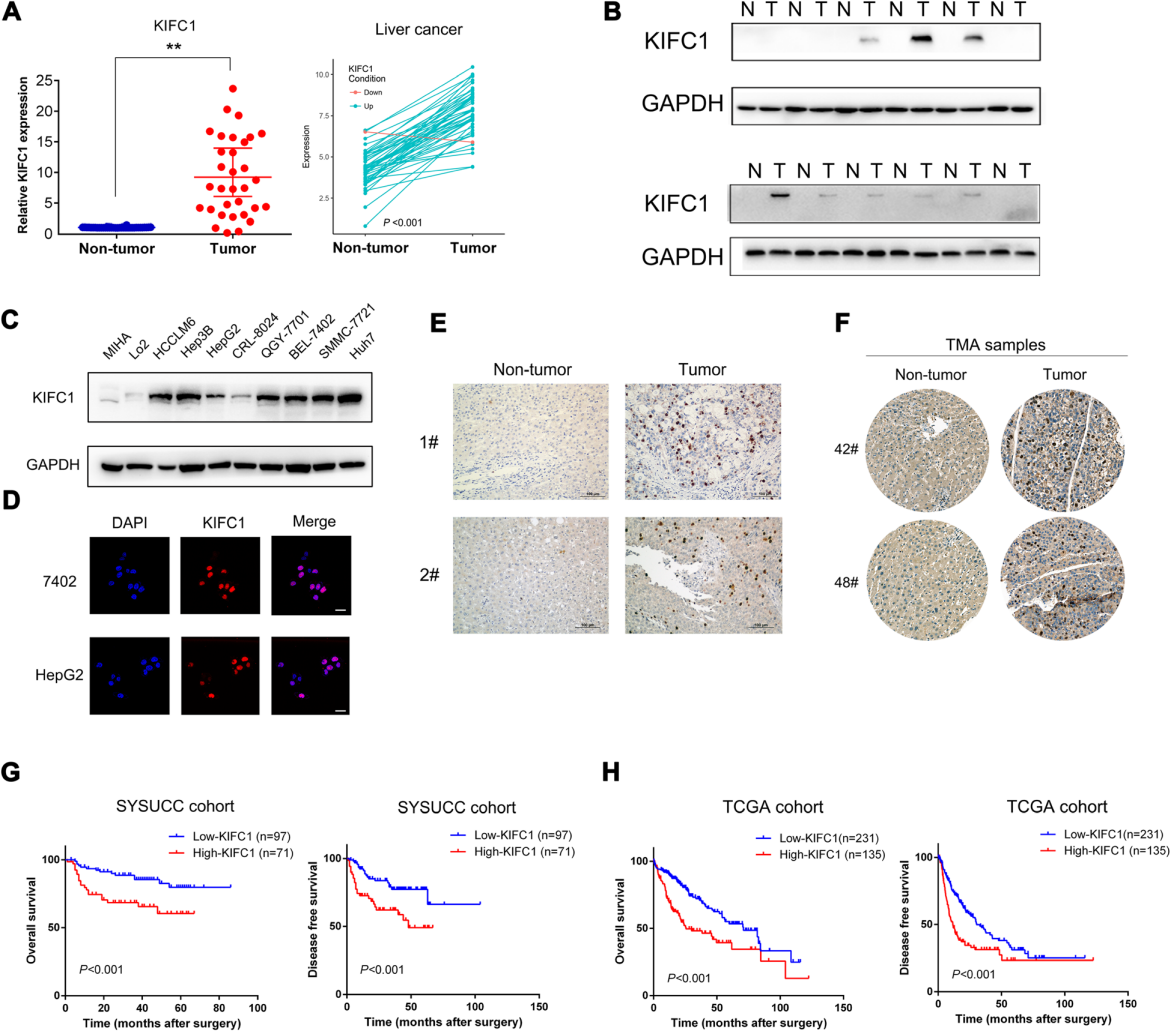
KIFC1 was identified as an oncogenic factor in HCC and is associated with poor survival and advanced stages. **a** The fold change of KIFC1 mRNA expression in 40 paired HCC and adjacent nontumor tissues and liver cancer dataset from TCGA database. Data are presented as the mean ± SD, * *P* < 0.01, ** *P* < 0.001. **b** KIFC1 protein expression in 12 pairs of HCC and adjacent nontumor tissues. **c** Expression of KIFC1 in HCC cell lines (HepG2, Hep3B, QGY-7701, BEL-7402, SMMC-7721, Huh-7, CRL-8024, and LM6) and immortalized hepatocytes MIHA and LO2 were examined by western blotting. **d** and **e**: IF and IHC assays demonstrated that KIFC1 was mainly located in the nucleus in both HCC cell lines and patient tissues.The bar indicated 10 μm. **f** Representative images of KIFC1 expression in TMA detected by IHC from both adjacent nontumor and HCC tissues. **g** Kaplan-Meier analysis indicated the correlation between high expression of KIFC1 and poor OS and DFS rates in HCC patients. **h** Survival curves from TCGA cohort validated that HCC patents with high levels of KIFC1 had shortened OS and DFS rates

The expression of KIFC1 in HCC and adjacent normal tissue samples was tested by both qRT-PCR and western blotting. KIFC1 was mainly overexpressed in HCC samples, and this was validated by data from TCGA database (https://cancergenome.nih.gov/) (Fig. 1a and b). The expression results from ten liver cell lines revealed that KIFC1 was highly expressed in most HCC cells and it’s expression in MIHA and LO2, which belongs to the normal human hepatic cell line, was low. HepG2 and 8024 cells had relatively low levels of KIFC1, while 7701 and 7402 had relatively high levels of KIFC1. Thus, they were selected to construct KIFC1 overexpression and knockdown cells (Fig. 1c). Further immunofluorescence and immunohistochemistry analyses revealed that KIFC1 is located mainly in the nucleus (Fig. 1d and e).

### KIFC1 is positively associated with advanced clinical stages and poor prognosis in HCC patients

To investigate the potential clinical and prognostic significance of KIFC1 expression in HCC patients, we conducted IHC staining for KIFC1 in 168 formalin-fixed and paraffin-embedded (FFPE) HCC specimens and 30 paired adjacent normal liver tissues. KIFC1 expression in all the adjacent normal tissues was absent or at low levels, whereas 71/168 (42.3%) of the HCC specimens had high levels of KIFC1 (Fig. 1f). Correlation analysis revealed that KIFC1 overexpression was positively associated with advanced stages, tumor size and recurrence (*P* < 0.05, Table 1). The overall survival (OS) and disease-free survival (DFS) rates illustrated by Kaplan-Meier analysis showed that patients with higher levels of KIFC1 had shorter survival times. The mean survival time for HCC patients with low levels of KIFC1 was 74.15 months compared with 45.57 months for patients with high levels of KIFC1 expression (*P* < 0.002, log-rank test, Table 2). Both OS and DFS survival rates were further confirmed by data from TCGA cohort (*P* < 0.001, Fig. 1g and h). Further multivariate Cox regression determined that KIFC1 was an independent prognostic factor for the poor survival of HCC patients (RR 2.371, 95% CI 1.207 to 4.660, *P* = 0.012, Table 2).

**Table 1:** Correlation expression of KIFC1 and clinicopathological parameters in 168 HCC cases in SYSUCC cohort

Variable	All cases (*N* = 168)	KIFC1 expression (%)		*P* values
		Low expression (*N* = 97)	High expression (*N* = 71)	
Age (years)				0.763
≤51	90	51 (56.7)	39 (43.3)	
>51	78	46 (59.0)	32 (41.0)	
Sex				0.849
Male	143	83 (58.0)	60 (42.0)	
Female	25	14 (56.0)	11 (44.0)	
AFP (ng/ml)				0.652
≤20	72	43 (59.7)	29 (40.3)	
>20	96	54 (56.3)	42 (43.7)	
HBsAg				0.394
negative	11	5 (45.5)	6 (54.5)	
positive	157	92 (58.6)	65 (41.4)	
Cirrhosis				0.713
No	99	56 (56.6)	43 (43.4)	
Yes	69	41 (59.4)	28 (40.6)	
Necrosis				0.506
No	76	46 (60.5)	30 (39.5)	
Yes	92	51 (55.4)	41 (44.6)	
Clinical stage				**0.009**
-	111	72 (64.9)	39 (35.1)	
-	57	25 (43.9)	32 (56.1)	
Histologic grade (WHO)				0.113
G1-G2	83	53 (63.9)	30 (36.1)	
G3-G4^a^	85	44 (51.8)	41 (48.2)	
Tumor size (cm)				**0.031**
≤5	73	49 (67.1)	24 (32.9)	
>5	95	48 (50.5)	47 (49.5)	
Tumor multiplicity				0.688
Unifocal	128	75 (58.6)	53 (41.4)	
Multifocal	40	22 (55.0)	18 (45.0)	
Intravascular emboli				0.688
No	100	59 (59.0)	41 (41.0)	
Yes	68	38 (55.9)	30 (44.1)	
Recurrence				**0.037**
No	125	78 (62.4)	47 (37.6)	
Yes	43	19 (44.2)	24 (55.8)	

^a^:including 3 cases sarcomatoid liver cancer

*P* values less than 0.05 are in boldface

**Table 2:** Univariate and multivariate analysis of different prognostic parameters in 168 HCC patients

Variable	Univariate analysis			Multivariate analysis	
	All cases	Mean survival (months)	*P* Values	HR (95% CI)	*P *Values
Age (years)			0.339		
≤51	90	49.799			
>51	78	69.089			
Sex			0.061		
Male	143	69.106			
Female	25	43.696			
AFP (ng/ml)			0.055		
≤20	72	72.295			
>20	96	53.431			
HBsAg			0.329		
negative	11	59.111			
positive	157	66.402			
Cirrhosis			0.829		
No	99	57.617			
Yes	69	66.272			
Necrosis			0.179		
No	76	60.350			
Yes	92	63.514			
Clinical stage			**0.000**		**0.000**
-	111	65.813		1	
-	57	43.778		5.173 (2.456–10.894)	
Histologic grade (WHO)			**0.000**		**0.035**
G1-G2	82	65.254		1	
G3-G4^a^	86	55.882		2.334 (1.063–5.126)	
Tumor size (cm)			**0.017**		0.915
≤5	73	74.428			
>5	95	49.049			
Tumor multiplicity			**0.000**		0.478
Unifocal	128	61.089			
Multifocal	40	50.483			
Intravascular emboli			**0.013**		0.483
No	100	61.257			
Yes	68	59.172			
Recurrence			**0.007**		0.061
No	125	70.974			
es	43	44.667			
KIFC1			**0.002**		**0.012**
Low		74.153		1	
High		45.568		2.371 (1.207–4.660)	

^a^:including 3 cases sarcomatoid liver cancer

*P* values less than 0.05 are in boldface

### KIFC1 supports HCC growth in vitro and in vivo

**Fig. 2:**
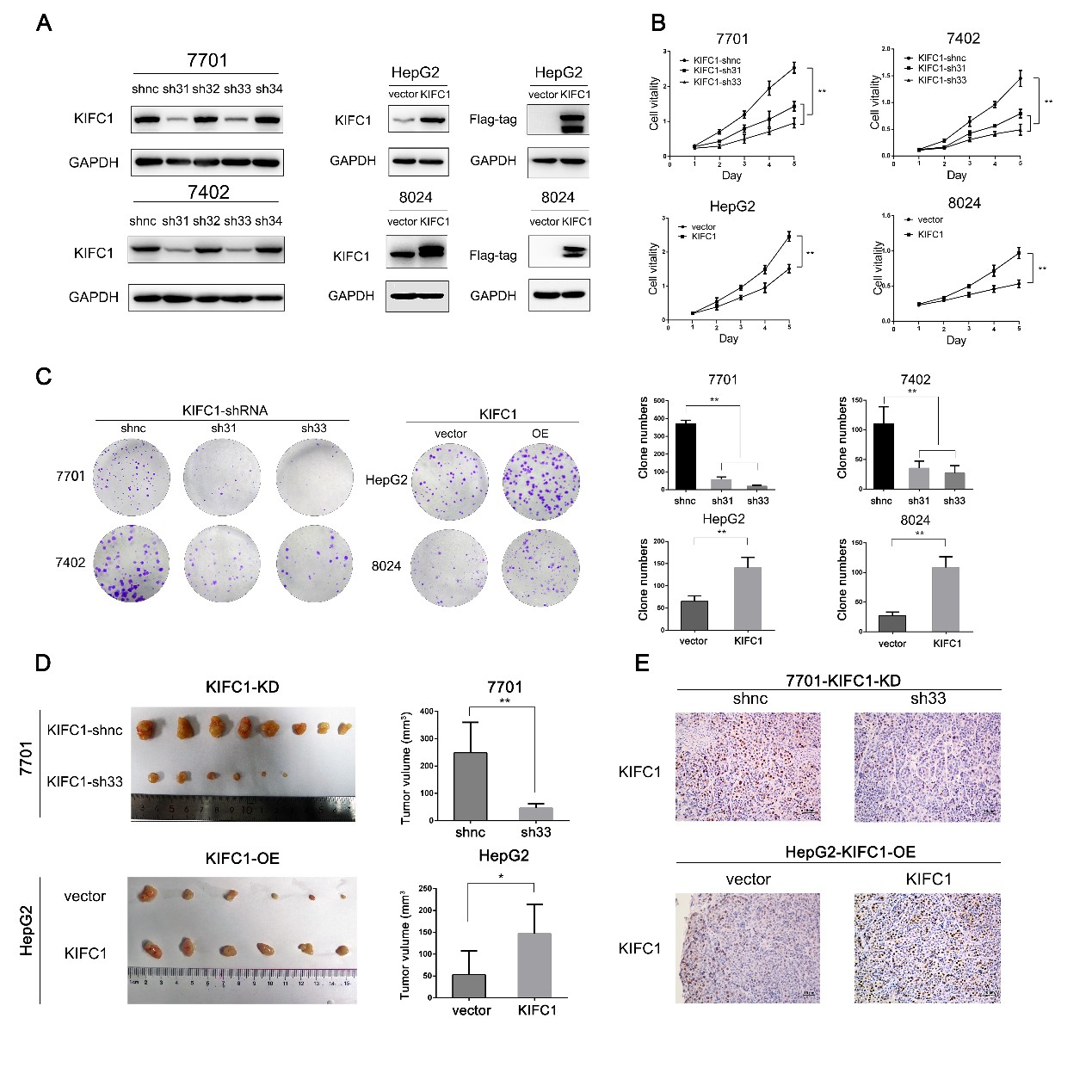
KIFC1 supports HCC cell proliferation in vitro and tumorigenicity in vivo. **a** Western blotting revealed that KIFC1 was efficiently knocked down in shRNA 31 (sh31) and shRNA 33 (sh33) and overexpressed in the corresponding cells. **b** The cell proliferation ability of the indicated cells was demonstrated by the CCK-8 assay. **c** Clone formation ability was tested in HCC cells with KIFC1 knockdown or overexpression. The clone numbers were counted and are presented in the right panels. Data are presented as the mean ± SD, * *P* < 0.01, ** *P* < 0.001. **d** Harvested xenografts formed by the KIFC1 knockdown 7701 and KIFC1 overexpression HepG2. Tumor volume was calculated and is presented in the right panels. Data are presented as the mean ± SD, * *P* < 0.01, ** *P* < 0.001. **e** Tissues from xenograft neoplasms by KIFC1 knockdown 7701 and KIFC1 overexpression HepG2 were tested by IHC for KIFC1 expression

To further investigate the potential effects of KIFC1 on HCC, shRNAs and an overexpression vector were used to establish KIFC1 knockdown and ectopic expression cells. Efficacy was confirmed by western blot. Among the four short hairpin RNAs tested, shRNA 31 (sh31) and shRNA 33 (sh33) demonstrated the most significant knockdown effect and were selected for subsequent experiments. The ectopic expression vector tagged with Flag was also constructed for further coIP assays (Fig. 2a). We performed CCK-8 and plate colony formation assays to assess the role of KIFC1 in HCC growth and proliferation. KIFC1 overexpression promoted HCC proliferation and foci formation. When KIFC1 was knocked down, proliferation and clone formation ability decreased (Fig. 2b and c). To validate the in vivo effect of KIFC1 on tumor growth, a tumor subcutaneous xenograft model was established. The tumor volume in the KIFC1 knockdown group was significantly less than that in the control group. The tumor volume in the KIFC1 overexpression group demonstrated the opposite results (Fig. 2d). The expression of KIFC1 in xenograft tumors was supported by IHC staining (Fig. 2e).

### KIFC1 promotes HCC invasion and metastasis in vitro and in vivo

**Fig. 3:**
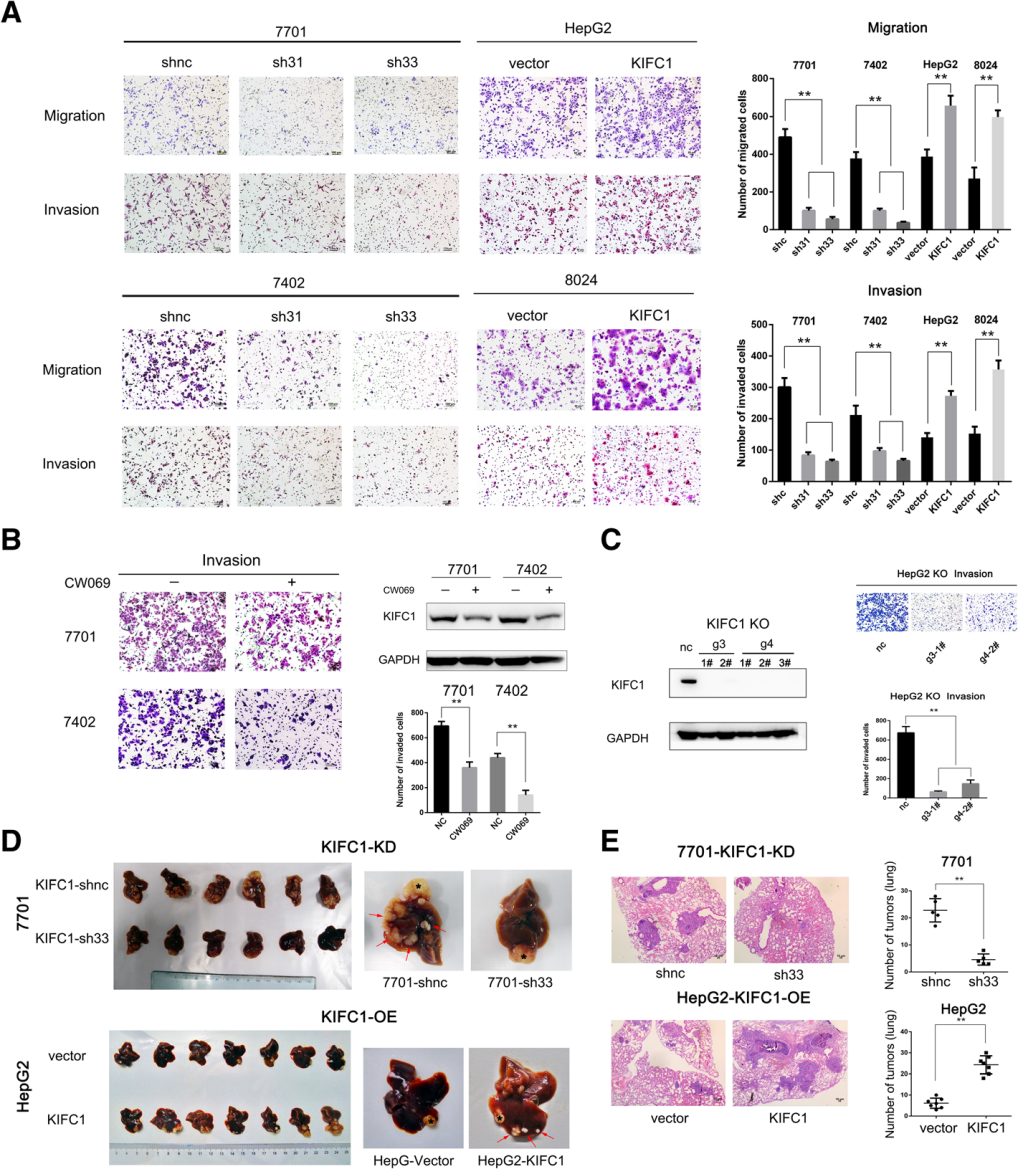
KIFC1 promotes HCC cell invasion in vitro and accelerates intrahepatic and lung metastasis in vivo. **a** Transwell migration and invasion assays demonstrated the migratory and invasive abilities of the indicated HCC cells. The counts of migrated and invaded HCC cells are illustrated in the right panels. Data are presented as the mean ± SD. * *P* < 0.01, ** *P* < 0.001. **b** Western blotting indicated the KIFC1 inhibition by CW069 (60 μM) for 48 h. Transwell invasion assays demonstrated the invasive ability of the indicated cells. **c** KIFC1 was knocked out with the CRISPR-Cas9 system as described in the protocol from Feng Zhang’s laboratory. Western blot demonstrated that KIFC1 was knocked out by gRNA sequence g3 and g4. Transwell invasive abilities were compared between the indicated HCC cells. **d** Harvested liver tissue from orthotopic implantation models revealed intrahepatic metastasis differences in the 7701 with KIFC1 knockdown (KD) or KIFC1 overexpression (OE) HepG2. Representative pictures of intrahepatic metastatic nodules are shown in the right panels. An asterisk marks the tumor developing at the injected site and an arrow indicated the intrahepatic metastatic lesions. **e** Metastasis lesions from the lung metastasis model are illustrated by HE staining. The counts are illustrated on the right panels. Data are presented as the mean ± SD. * *P* < 0.01, ** *P* < 0.001

KIFC1 interacts with microtubules to transport intracellular cargo. Microtubules play an important role in maintaining cytoskeletal homeostasis, which can contribute to cell migration and invasion [17]. We therefore evaluated the role of KIFC1 in regulating HCC invasion and metastasis. Both migratory and invasive abilities were markedly suppressed when KIFC1 was knocked down in 7701 and 7402 cells; however, highly expressed KIFC1 increased HCC migration and invasion in HepG2 and 8024 cells (Fig. 3a). A recent study reported that CW069 is a novel and allosteric inhibitor of KIFC1. It is highly selective and binds to the loop 5 cleft of KIFC1’s globular motor domain which disrupts KIFC1’s ability to drive the motility of microtubules [18]. We also tested the invasive ability with both KIFC1 inhibitor CW069 and KIFC1 knockout (KO) cells and those results confirmed that KIFC1 promoted HCC migration and invasion (Fig. 3b and c).

To investigate whether KIFC1 can regulate HCC metastasis in vivo, both orthotopic implantation and lung metastasis mouse models were constructed. For HCC orthotopic models, the tumor nodule number spread into the liver of the KIFC1 ectopic expression group was higher than that in the vector group. This regulation was confirmed in 7701 cells with low levels of KIFC1 (Fig. 3d). For the tail vein injection model, lung metastasis lesions in the KIFC1 knockdown group were smaller and fewer than those in the control group (Fig. 3e). Taken together, these results revealed that KIFC1 promoted HCC invasion and metastasis both in vitro and in vivo.

### KIFC1 induces epithelial-mesenchymal transition in HCC cells

**Fig. 4:**
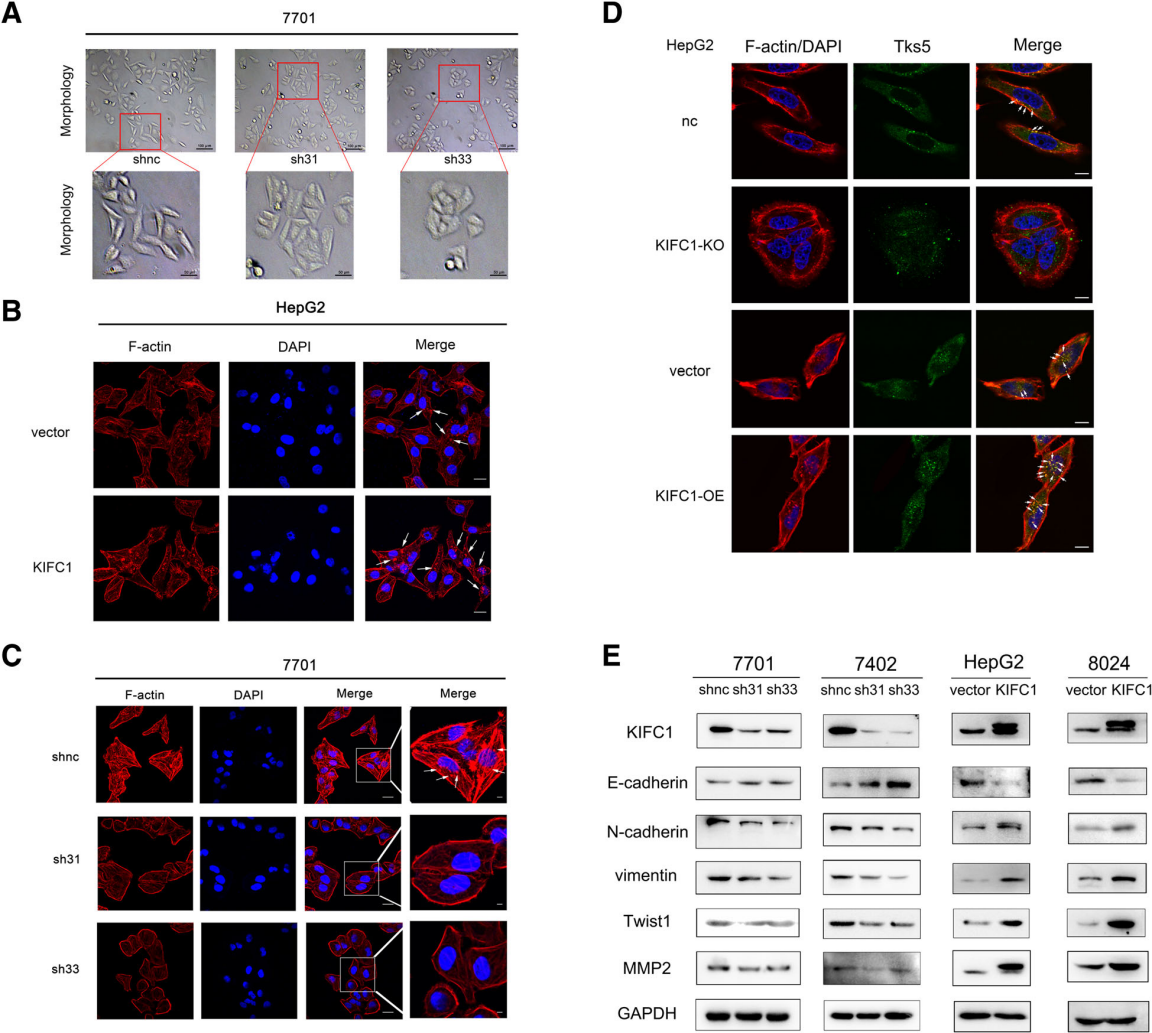
KIFC1 induces EMT in HCC cells. **a** Cell morphology changed from a spindle-like appearance to a cobblestone-like appearance in KIFC1-knockdown 7701 cells. **b** There were more pseudopodia formation in HepG2-KIFC1 cells compared with HepG2-vector cells. The bar indicated 40 μm (magnification: 600 ×). **c** Pseudopodia were formed in 7701-KIFC1-control cells and decreased in KIFC1-knockdown cells. The bar indicated 40 μm (magnification: 600 ×).**d** The colocalization of F-actin and Tks5 in KIFC1 knockout (KO) cells and KIFC1 overexpression (OE) cells. The white arrows indicated the colocalization sites. The bar indicated 40 μm (magnification: 600 ×). **e** In KIFC1-overexpressing cells, mesenchymal markers such as vimentin and Twist1 were increased, and the epithelial marker E-cadherin was downregulated

Epithelial-mesenchymal transition **(**EMT) is a key cellular process associated with invasion and metastasis in many cancers. It is associated with altered gene expression patterns resulting in the loss of E-cadherin and the breakdown of cell-cell junctions as well as the acquisition of a fibroblastic morphology, including polarized actin cytoskeleton assembly into protrusive and invasive pseudopodial structures [19]. A typical morphological change of EMT is from characteristic cobblestone-like epithelial morphology to a spindle, fibroblast-like shape [20, 21]. KIFC1 knockdown facilitated HCC cell change from a spindle to a cobblestone-like appearance, which indicated that KIFC1 promoted EMT change (Fig. 4a). It has been suggested that invadopodia are specialized membrane protrusions possessing protease activity that participate in cancer cell invasion. We next stained F-actin with rhodamine-conjugated phalloidin to study the reorganization of the cytoskeleton. F-actin staining was reduced, and fewer pseudopodia were observed in KIFC1-knockdown 7701 cells. However, pseudopodia were increased in KIFC1-overexpressing HepG2 cells (Fig. 4b and c). Tks5 (tyrosine kinase substrate with five SH3 domains, also known as FISH), a Src substrate, is the master scaffold protein for invadopodia formation and function [22]. As Tks5 does not seem to localize to other actin-based structures, such as filopodia and lamellipodia, the colocalization of F-actin and Tks5 with focal ECM degradation can be used to identify invadosome such as invadopodia [23]. Here we demonstrated that the colocalization of F-actin and Tks5 was increased in KIFC1 overexpression HepG2 cells, which indicated the upregulation of invadopodia formation (Fig. 4d). We further evaluated EMT markers by western blotting. The epithelial marker E-cadherin was increased, whereas the mesenchymal markers Twist1 and vimentin were downregulated in KIFC1-knockdown 7701 and 7402 cells. The EMT change was also confirmed in KIFC1-overexpressing HepG2 and 8024 cells (Fig. 4e).

The MAPK/Erk and Jak/Stat pathways have been implicated in HCC pathogenesis [24, 25]. Key molecules in those pathways were tested, and phosphorylated Stat3 was elevated in high-level KIFC1 HCC, which indicated that KIFC1 overexpression activated the Jak/Stat pathway (Additional file 3: Figure S1). Taken together, KIFC1 promoted HCC invadopodia formation, facilitated EMT and activated the Jak/Stat pathway.

### KIFC1 interacts with the transcriptional factor HMGA1 by coimmunoprecipitation

**Fig. 5:**
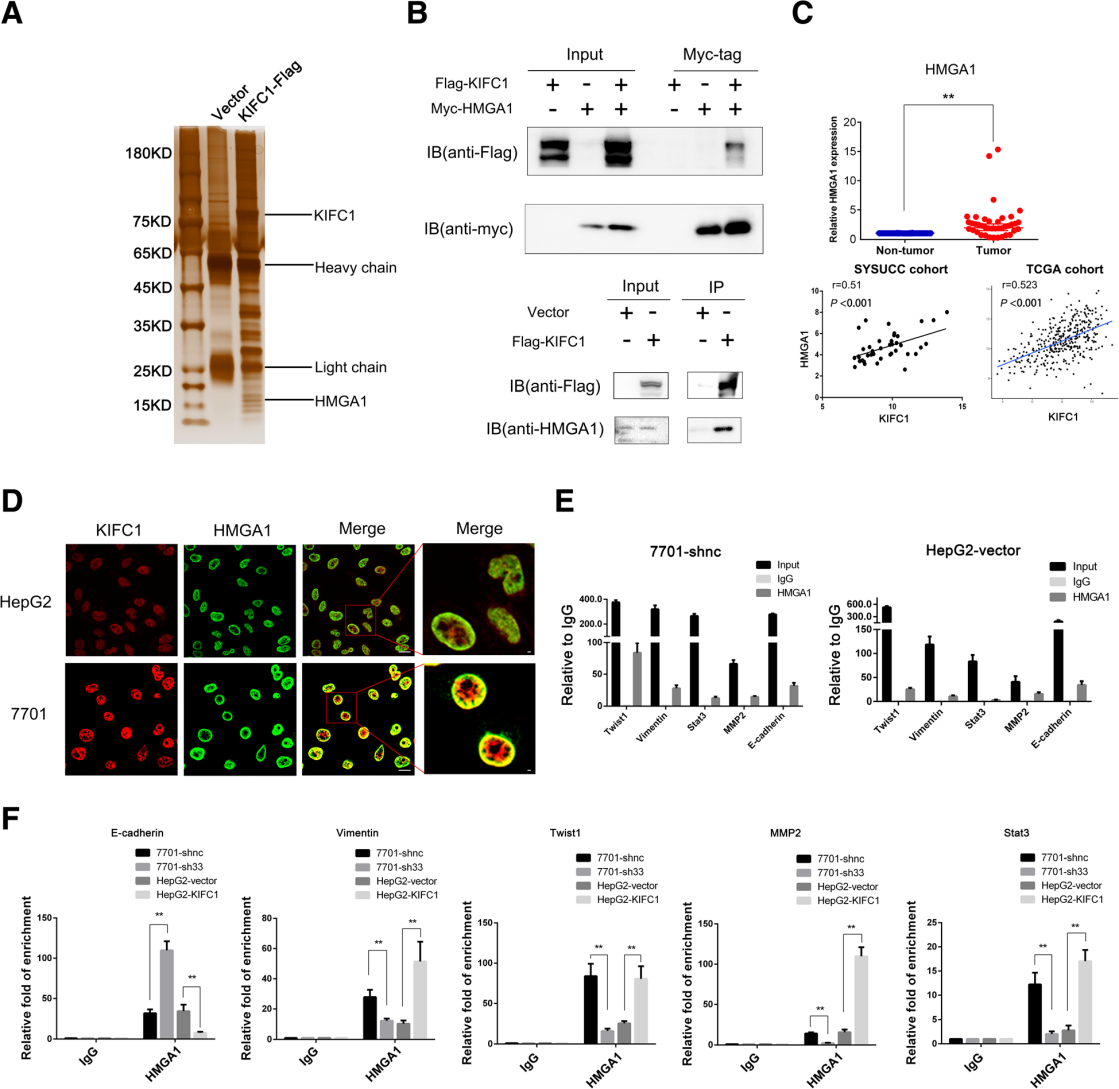
KIFC1 interacts with HMGA1 and regulates its transcriptional activity in HCC cells. **a** A silver staining assay was used to distinguish differentiated proteins between control and KIFC1-overexpressing HepG2 cells. HMGA1 (17 kD) is indicated by a black bar. **b** HEK-293 T cells were transfected with Flag-KIFC1 or Myc-HMGA1 or both vectors. A coIP assay was performed to test the interaction (upper part). HepG2-vector and HepG2-KIFC1-Flag cells were also tested by the coIP assay (lower part). Flag-tag antibody (14793 s, Cell signaling technology, 1:1000) and Myc-tag antibody (M192-3B, MBL, 1:1000) were used in the upper part. Flag tag antibody and HMGA1 (ab129153, Abcam, 1:1000) were used in the lower part. **c** The fold change in HMGA1 mRNA expression levels in 40 paired HCC and adjacent nontumor tissues. The correlation between KIFC1 and HMGA1 was tested in 40 paired specimens and data from TCGA cohort. Data are presented as the mean ± SD, * *P* < 0.01, ** *P* < 0.001. **d** IF assay demonstrated the colocalization of KIFC1 and HMGA1 in HepG2 and 7701 cells. The bar indicated 40 μm. **e** ChIP assay was performed using HMGA1 antibody (ab129153, Abcam). IgG was used as the negative control. Quantitative PCR was conducted at the promoter regions of Twist1, vimentin, Stat3, MMP2, and E-cadherin in 7701-shnc and HepG2 vector cells. **f** Binding levels of HMGA1 and E-cadherin, vimentin, Twist1, MMP2 and Stat3 promoters after KIFC1 knockdown or overexpression

We next tried to shed light on the mechanism by which KIFC1 promotes HCC pathogenesis. To identify the potential binding partners of KIFC1, whole cell protein lysates from HepG2-vector and HepG2-KIFC1-Flag cells were assayed for immunoprecipitation with a Flag-tag antibody. Silver staining was further conducted to distinguish the differential protein bands in the resultant immunoprecipitates (Fig. 5a). These immunoprecipitates were next analyzed by mass spectrometry (MS). The unique proteins of KIFC1-overexpressing cells were compared with another KIFC1 interaction protein dataset by Hein MY et al. [26] by MS. HMGA1, an architectural transcriptional factor, was the common protein between the two datasets (MS original data in Additional file 2). The results from further coimmunoprecipitation (coIP) and immunofluorescence assays validated this interaction (Fig. 5b and d). Thus, we wondered whether KIFC1 promotes HCC pathogenesis by regulating HMGA1.

### KIFC1 regulates HMGA1 transcriptional activity in HCC cells

HMGA1 was highly expressed in HCC tumor samples compared with adjacent nontumor samples in our clinical dataset. Furthermore, linear regression was used to uncover the correlation between KIFC1 and HMGA1. HMGA1 was positively correlated with KIFC1 expression (r = 0.51, *P* < 0.001), in accordance with data from TCGA cohort (Fig. 5c).

Through binding to nuclear chromatin at AT-rich regions with other transcription factors, HMGA1 has the ability to alter chromatin structure and regulate gene transcription. We next tested several key genes involved in HCC proliferation and metastasis. HMGA1 binds to the promoter regions of Stat3, MMP2 and EMT-related genes E-cadherin, vimentin, and Twist1 and modulates their transcription (Fig. 5e). Furthermore, the mRNA levels of vimentin, Twist1, MMP2, and Stat3 by ChIP-PCR were elevated in KIFC1 ectopic expression HCC compared with KIFC1-knockdown HCC. E-cadherin was decreased in the comparison (Fig. 5f). These results indicated that KIFC1 interacted with HMGA1 and regulated its transcriptional activity.

### HMGA1 is responsible for KIFC1-enhanced HCC cell proliferation and invasion

**Fig. 6:**
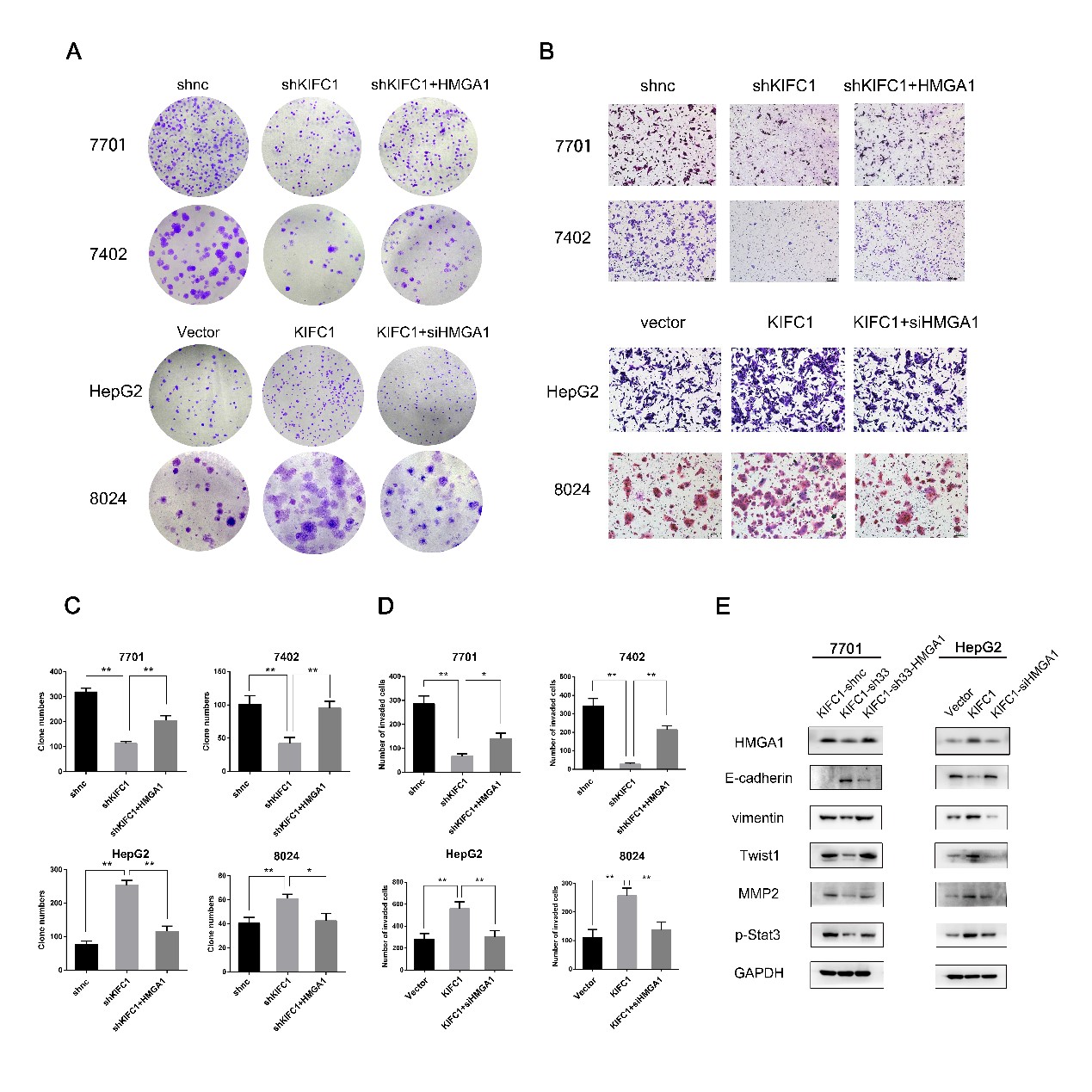
HMGA1 is responsible for KIFC1-enhanced HCC proliferation and invasion. **a** and **c** HMGA1 restored clone formation inhibited by KIFC1 knockdown. The counts of clone cell numbers are shown in panel **b**. shKIFC1 was used the same sequences as sh33 (5′- ccagggctatcaaataaagaa-3′). Data are presented as the mean ± SD, * *P* < 0.01, ** *P* < 0.001. **b** and **d** HMGA1 restored the invasive ability inhibited by KIFC1 knockdown. The counts of invaded cell numbers are shown in panel **d**. Data are presented as the mean ± SD, * *P* < 0.01, ** *P* < 0.001. **e** Western blot assay indicated that the levels of mesenchymal markers (vimentin, Twist1) as well as MMP2, p-Stat3 decreased while the level of epithelial marker E-cadherin increased in KIFC1 knockdown 7701 cells. This phenomenon could be reversed by enforced expression of HMGA1 in 7701 KIFC1 knockdown cells

To investigate the function of HMGA1 in KIFC1-enhanced HCC proliferation and invasion, HMGA1 was overexpressed in KIFC1-knockdown cells, while it was interfered with RNAi in KIFC1-overexpressing HCC (RiboBio, Guangzhou, China). Clone forming assays revealed that HMGA1 knockdown reversed KIFC1-induced clone formation. In addition, the invasive ability of KIFC1-highly expressed HCC could also be reversed by HMGA1 interference (Fig. 6a-d). Meanwhile, HMGA1 was decreased in KIFC1 knockdown 7701 cells and enforced HMGA1 expression rescued the decrease of vimentin, Twist1, MMP2 and p-Stat3 in KIFC1-silenced 7701 cells (Fig. 6e). HMGA1 facilitated the proliferation and invasive ability of KIFC1-highly expressed HCC by regulating EMT and Jak/Stat pathway.

### KIFC1 is activated by the Wnt/β-catenin pathway

**Fig. 7:**
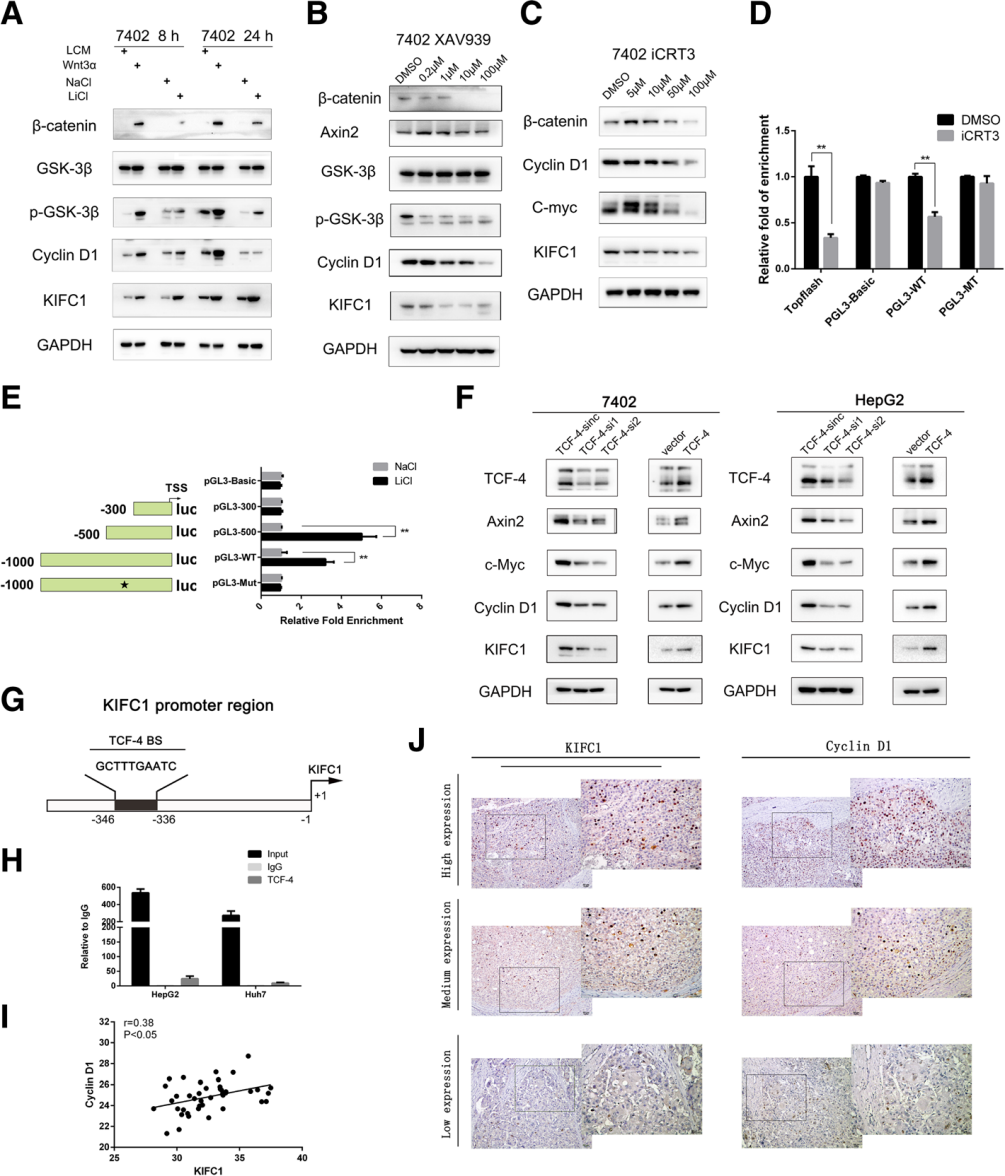
KIFC1 is activated by the Wnt/β-catenin pathway. **a** Western blot analysis of the expression of KIFC1 and Wnt/β-catenin pathway components in 7402 cells treated with Wnt3α medium or LiCl (30 mM) for the indicated times. **b** and **c** Western blot analysis revealed the expression of KIFC1 and Wnt/β-catenin pathway components with different doses of inhibitors. XAV939 inhibits tankyrase, and tankyrase is an enzyme that mediates the poly-ADP-ribosylation of Axin. Axin subsequently undergoes degradation. ICRT3 combines with β-catenin and inhibits its combination with TCF-4. Both inhibitors inhibit Wnt/β-catenin pathway activity. **d** A dual-luciferase assay showed the transcriptional activities regulated by iCRT3 treatment in the indicated KIFC1 wild type and mutant promoters. Data are presented as the mean ± SD, * *P* < 0.01, ** *P* < 0.001. **e** A dual-luciferase assay revealed the different transcriptional activities regulated by LiCl treatment in the indicated KIFC1 truncation, wild type and mutant promoters. The KIFC1 promoter sequences were mutated from 5′-GCTTTGAATC-3′ to 5′- TAAAAATCGT − 3′. Data are presented as the mean ± SD, * *P* < 0.01, ** *P* < 0.001. **f** Western blot analysis revealed the expression of KIFC1 and other TCF-4 regulating genes in TCF-4-silenced and TCF-4 overexpression 7402 and HepG2 cells. **g** Illustration of predicted binding sites from the ALGGEN-PROMO database. **h** ChIP assay was performed with TCF-4 antibody (2565, Cell signaling technology) IgG was used as the negative control. Quantitative PCR was conducted at the promoter regions of KIFC1 with HepG2 and Huh7 cell lines. **i** The correlation between KIFC1 and Cyclin D1 was performed in 40 HCC samples with qRT-PCR. **j** KIFC1 and Cyclin D1 expression in the same HCC sample was tested with IHC assay

To explore the upstream regulation of KIFC1, the ALGGEN-PROMO database (http://alggen.lsi.upc.es/cgi-bin/promo_v3/promo/promoinit.cgi?dirDB=TF_8.3) was used to search for potential transcriptional factors (TFs) that regulate KIFC1 expression. TCF-4, a key transcriptional factor participating in the Wnt/β-catenin pathway, was predicted to regulate KIFC1 transcription. The Wnt/β-catenin pathway is frequently activated in HCC and is implicated in many HCC events, including the pathogenesis of HCC, drug resistance and tumor progression [27]. Cell medium from the Wnt3-expressing L cell line was collected as reported previously [28]. Lithium chloride (LiCl) is an agonist of the canonical Wnt/β-catenin pathway that can inhibit GSK3β activity and thereby stabilize free cytosolic β-catenin effectively [29]. Both Wnt3α medium and LiCl administration served as agents to stimulate the Wnt/β-catenin pathway [30]. Another two agents, XAV939 and iCRT3, were used to inhibit the Wnt/β-catenin pathway. XAV939 is a potent, small molecule inhibitor of tankyrase (TNKS). By inhibiting TNKS activity, XAV939 stabilizes axin, increases the levels of the axin-GSK3β complex and promotes the degradation of β-catenin, thus inhibiting Wnt pathway [31]. iCRT3 acts as a selective β-catenin responsive transcription inhibitor by interfering with β-catenin-TCF4 interaction via direct β-catenin binding [32]. The results from both activation and inhibition experiments validated that activation of the Wnt/β-catenin pathway promoted KIFC1 expression (Fig. 7a-c). To explore whether this activation was regulated by TCF-4, a dual-luciferase reporter assay was used. The truncated reporter results indicated that the TCF-4 binding site was between − 300 bp and − 500 bp, in accordance with the binding site predicted by ALGGEN-PROMO (Fig. 7e and g). After mutation, luciferase activity was attenuated compared with wild-type in both the Wnt/β-catenin activation and inhibition models (Fig. 7d and e). In addition, the combination was further tested with ChIP-PCR assay with HepG2 and Huh7 (Fig. 7h). TCF-4 could bind to the KIFC1 promoter. Cyclin D1 was the downstream target gene of TCF-4. The mRNA expression of KIFC1 was positively correlated with Cyclin D1 in 40 HCC patients by qRT-PCR (Fig. 7i) and this positive correlation was further tested in HCC samples with IHC (Fig. 7j). These results demonstrate that KIFC1 is regulated by TCF-4 and that Wnt/β-catenin pathway activation promotes KIFC1 transcription.

### KIFC1 inhibition increases HCC cell sensitivity to paclitaxel

**Fig. 8:**
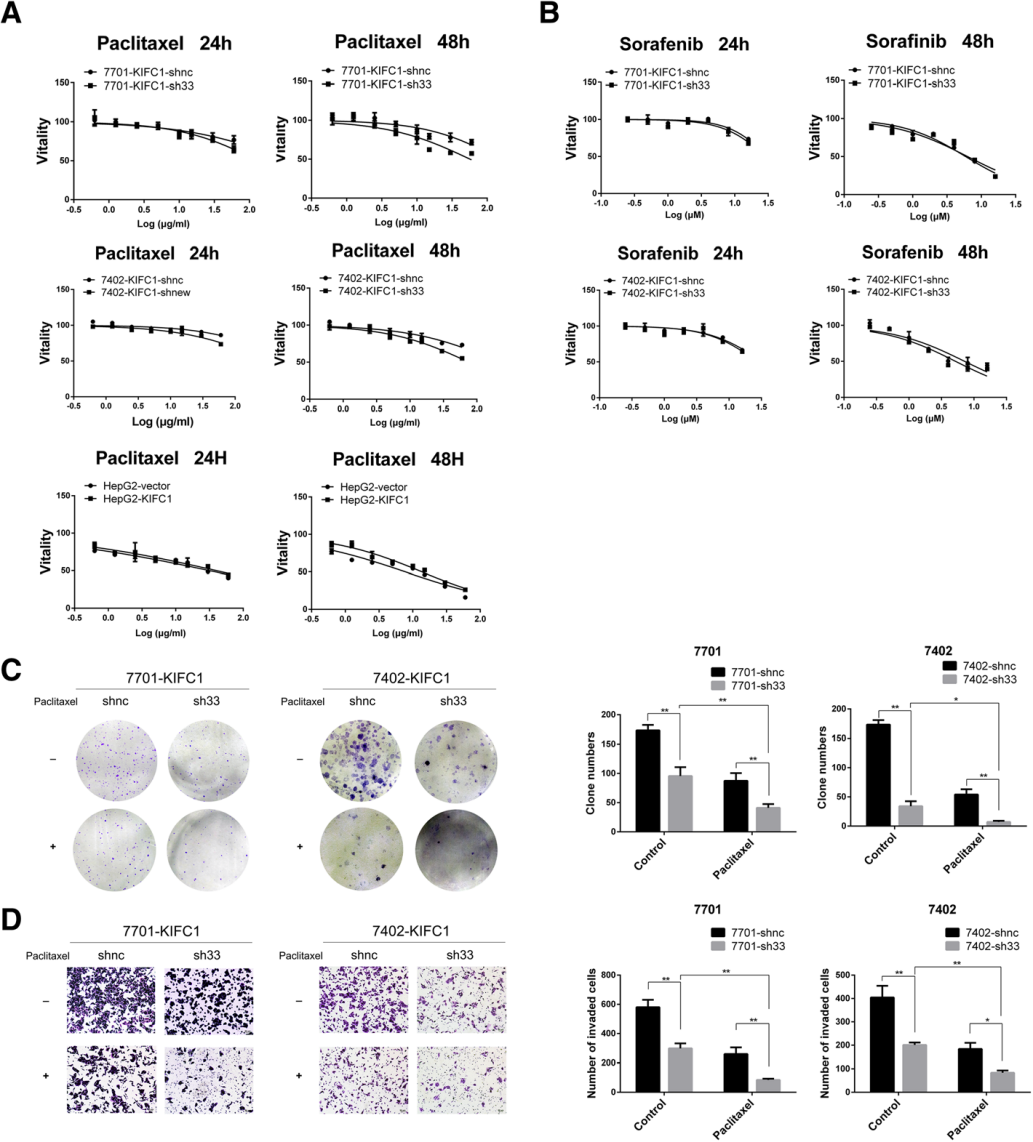
KIFC1 inhibition enhances HCC cell sensitivity to paclitaxel. **a** and **b** Growth curves depicted from the CCK-8 assay revealed cell vitality following treatment with paclitaxel and sorafenib. Paclitaxel was tested in KIFC1-knockdown 7701 and 7402 cells and KIFC1-overexpressing HepG2 cells. Sorafenib was examined in KIFC1-knockdown 7701 and 7402 cells. **c** and **d** Clone formation and Transwell invasion assays were tested with or without paclitaxel in the indicated cells. The counts of clone and invaded cell numbers are shown in the right panels. Data are presented as the mean ± SD, * *P* < 0.01, ** *P* < 0.001

Studies on breast cancer, prostate cancer and HCC have suggested that kinesin family proteins, including KIFC1, are responsible for drug resistance [7, 10, 33]. Since KIFC1 expression is usually elevated in HCC, we wondered whether KIFC1 knockdown could increase HCC cell sensitivity to paclitaxel. The IC50/48 h values were 152.60 μg/ml and 57.22 μg/ml in KIFC1 control and knockdown 7701 cells, respectively. The cell vitality change was not as significant in the KIFC1 overexpression group (7.73 μg/ml vs 13.66 μg/ml, 48 h) compared with the KIFC1 knockdown group (152.60 μg/ml vs 57.22 μg/ml, 48 h) (Fig. 8a and b, Table 3). KIFC1 inhibition provided a synergistic effect to increase HCC cell sensitivity to paclitaxel. This sensitivity enhancement was further validated by clone formation and invasion assays (Fig. 8c and d). Sorafenib was the first-line systemic treatment for HCC patients with advanced stages. When administered with KIFC1 control and knockdown cells, the IC50 value changed slightly (7701 48 h control vs knockdown = 5.93 μM vs 6.32 μM, Fig. 8b, Table 3). Paclitaxel, rather than sorafenib, seemed to be feasible to combine with a KIFC1 inhibitor for further HCC treatment. This result provides clinicians with new therapeutic insight into the treatment of KIFC1-overexpressing HCC patients.

**Table 3:** The IC50 for paclitaxel and sorafenib

		IC50	
Drug	Cell type	24 h	48 h
Paclitaxel (μg/ml)	7701-KIFC1-shnc	370.70	152.60
	7701-KIFC1-sh33	132.30	57.22
	7402-KIFC1-shnc	679.40	213.7
	7402-KIFC1-sh33	334.80	75.81
	HepG2-vector	31.45	7.73
	HepG2-KIFC1	39.84	13.66
Sorafenib (μM)	7701-KIFC1-shnc	31.02	5.93
	7701-KIFC1-sh33	29.25	6.32
	7402-KIFC1-shnc	32.80	7.19
	7402-KIFC1-sh33	27.76	5.21

## Discussion

As we performed this research, two studies sequentially reported that KIFC1 served as an HCC prognostic factor and promoted HCC proliferation and invasion [34, 35]. These studies, together with our data from the SYSUCC cohort, confirm that KIFC1 plays an important role in HCC pathogenesis. Regarding the underlying molecular mechanisms, Fu et al. did not conduct further exploration, and Han et al. revealed that KIFC1 was regulated by miR-532-3p and promoted HCC metastasis by gankyrin/AKT signaling. Here, we found a novel mechanism and demonstrated the clinical translation prospect of KIFC1 inhibition for HCC therapy.

KIFC1 is one minus end-directed kinesin of the kinesin-14 family. In this study, we found that 42.3% of patients in our SYSUCC cohort had high KIFC1 expression levels, while almost no KIFC1 expression was detected in adjacent normal tissue. KIFC1 was highly expressed in HCC compared with adjacent nontumor samples. These results indicate that KIFC1 might play important roles in cancer carcinogenesis and progression.

As a characteristic of cancer, centrosome amplification is often observed in HCC [36]. KIFC1 is involved in clustering extra centrosomes and securing bipolar spindles during mitosis [3]. This process is dynamic, during which KIFC1 is transported into the nucleus by importin alpha/beta and Ran-GTPase through interactions with undefined nucleoporin complexes [37, 38]. When fulfilling its functions, KIFC1 is degraded by E3 ligase APC/C through ubiquitination in the late metaphase. One interesting phenomenon we found was that KIFC1 expression in HCC cells appears heterogeneous, high in some cells and low in others (Fig. 1d). During the cell cycle, the cycle phases may vary among different cells. The KIFC1 expression also vary with the cycle phases and this may explain the heterogeneous expression of KIFC1 among HCC cells from the same cell line. In addition, the existence of KIFC1 expression allows HCC cells to avoid multipolar division and promotes proliferation. For a normal cell, there is no extra centrosome, and KIFC1 is normally a nonessential kinesin motor. These findings might explain why KIFC1 was mainly located in the nucleus and overexpressed in HCC compared with adjacent nontumor tissue.

Patients with high KIFC1 expression in our cohort had both shortened overall survival and disease-free survival, in accordance with data derived from the TCGA cohort. The univariate and multivariate survival analyses revealed that KIFC1 overexpression was associated with advanced stages and a short survival time. All of these factors make KIFC1 a crucial prognostic indicator for HCC.

The results from our HCC cell assays and mouse model demonstrated that KIFC1 overexpression promoted tumor proliferation and metastasis. As we discussed above, KIFC1 is essential for bipolar spindle formation, and KIFC1 knockdown induced multipolar spindle mitotic defects in cancer cells containing extra centrosomes and induced cancer cell death [39]. A recent study by Fu et al. also revealed that KIFC1 knockdown induced HCC cell apoptosis and cell death [34]. Both mitotic function and apoptosis contributed to the lower proliferation rate in KIFC1-knockdown HCC cells.

Regarding aggressiveness, studies have suggested that KIFC1 expression is associated with brain metastasis in primary non-small cell lung cancer [40]. However, the underlying molecular mechanisms were not illuminated.

In our KIFC1-knockdown HCC cells, we observed more cobblestone-like cells than spindle-like cells. The cobblestone-like appearance is commonly recognized as a characteristic of epithelial cells. Invadopodia are specialized membrane protrusions possessing protease activity that participate in cancer cell invasion. KIFC1-overexpressing HepG2 cells demonstrated more invadopodia than control cells. In addition, invadopodia expression was decreased when KIFC1 was knocked down.

KIFC1 promoted the expression of mesenchymal markers, such as vimentin and Twist1, as revealed by western blot. These results indicated that KIFC1 promoted HCC metastasis through EMT.

The regulation of the PI3K/AKT pathway by KIFC1 was reported to promote HCC proliferation and metastasis [34, 41]. Here, we found that the Jak/Stat pathway was also activated in HCC. Constitutive activation of Stat3 is involved in many cellular processes, including cell growth, metastasis, angiogenesis, and immune suppression, all of which favor HCC initiation and progression [25]. Our results showed that the Jak/Stat pathway was regulated by KIFC1 and provides another way to explain HCC pathogenesis.

HMGA1 is an architectural transcription factor. The subfamily consists of both the HMGA1a and HMGA1b protein isoforms (formerly HMG-I and HMG-Y), which result from the alternative splicing of HMGA1 mRNA. HMGA1 preferentially binds to the minor groove of chromatin at AT-rich regions and alters the DNA structure to facilitate the binding of other transcription factors and orchestrates the assembly of a transcriptional complex or “enhanceosome” to regulate the transcription of several genes by either enhancing or suppressing transcription factors [42, 43].

HMGA1 is involved in a variety of fundamental biological processes, ranging from embryonic development, cell proliferation, differentiation, senescence and metastasis [44, 45]. Numerous pieces of evidence have shown that HMGA1 is overexpressed in neoplasms, such as colon cancer [46], breast cancer [47], pancreatic cancer [48], cervical cancer [49] and liver cancer [50]. Studies have revealed that HMGA1 participates in HCC pathogenesis and predicts a poor prognosis in HCC patients [50, 51].

Our results revealed that HMGA1 was the binding protein of KIFC1. This result was in accordance with Maurizio’s discovery that KIFC1 interacted with HMGA1. KIFC1 expression was decreased in HMGA1-silenced breast cancer cells, while HMGA1 was decreased in KIFC1 knockdown HCC cells in our study [52]. This regulation was mutual and the combination might influence the stability of both genes. When one gene was silenced, another gene expression was decreased. As the case in HMGA1-stat3 regulation axis, HMGA1 induces stat3 expression and stat3 also feeds forward to upregulate HMGA1, leading to enhanced expression of both genes during tumor progression, thus form the so called ‘feed forward loop’ [53]. The interaction between KIFC1 and HMGA1 might share the similar regulation mechanism with HMGA1 and Stat3.

HMGA1 promoted HCC cell proliferation and metastasis. HMGA1 binds to the promoters of Stat3 MMP2 and EMT-related genes and regulates gene transcription. High levels of KIFC1 expression upregulated the transcription of mesenchymal markers, such as vimentin and Twist1, and decreased the transcription of the epithelial marker E-cadherin. The transcription of Stat3 and MMP2 was also elevated in KIFC1-overexpressing HCC. These results indicated that HMGA1 modulated Stat3 MMP2 and EMT-related gene transcription and that high levels of KIFC1 regulated HCC proliferation and invasion by modulating HMGA1 transcriptional activity. Our results provide new insight into the regulatory mechanism of KIFC1 in HCC pathogenesis.

The Wnt/β-catenin pathway is commonly activated in HCC [27]. When extracellular Wnt ligands bind to the Frizzled receptor and the LRP 5/6 coreceptor, the destruction complex (APC, Axin and GSK-3) is dissolved, and β-catenin is released into the cytoplasm and translocated into the nucleus, where it binds to the TCF/LEF complex. This complex then promotes the transcription of target genes, such as Axin 2, Myc and cyclin D1, which are involved in cell proliferation, cell cycle regulation and metastasis [27, 54]. Our results demonstrated that TCF-4, an essential transcriptional factor participating in the Wnt/β-catenin pathway, binds to the promoter of KIFC1 and promotes KIFC1 transcription. This result provides an explanation for KIFC1 overexpression in HCC cells. KIFC1 was activated by the Wnt/β-catenin pathway and further promoted HCC pathogenesis.

Paclitaxel (Taxol) is a phytochemical compound isolated from *Taxus brevifolia* and has shown antitumor activity in a variety of neoplasms. Currently, paclitaxel is clinically employed as the first-line treatment for breast cancer, lung cancer, esophageal cancer, colon cancer, lymphoma, acute leukemia, ovarian cancer and gastric cancer [55]. However, the effects of paclitaxel are limited in HCC, and HCC is resistant to paclitaxel treatment [56].

Paclitaxel binds to the beta subunit of tubulin to prevent the dissociation of microtubules and disrupts their proper organization and elongation. KIFC1 crosslinks and slides microtubules, thereby producing forces that aid in supernumerary centrosome clustering. KIFC1 is nonessential in normal cells. However, it is essential for the viability of cancer cells with extra centrosomes. This makes KIFC1 an optimal tumor-selective drug target. Studies have demonstrated that breast cancer with high levels of kinesin is resistant to docetaxel [57]. This result made us wonder whether KIFC1 inhibition could be used together with paclitaxel to enhance the sensitivity of paclitaxel in HCC cells. Our results indicated that KIFC1 inhibition had a synergistic effect with paclitaxel to increase HCC drug sensitivity. Sorafenib, the first-line therapy for advanced-stage HCC, was mildly altered by KIFC1 inhibition. These results indicate that KIFC1 has clinical translation prospects in HCC treatment.

In summary, this study determined that KIFC1 was elevated in HCC and that high-level KIFC1 was associated with poor survival and advanced stages. KIFC1 overexpression activated the Stat3 pathway and EMT, thus promoting HCC proliferation and metastasis. Furthermore, we found that HMGA1 was the binding partner of KIFC1, which modulated the transcription of Stat3, MMP2 and EMT-related genes. High levels of KIFC1 promoted the binding ability of HMGA1 to the promoters of Stat3, MMP2, vimentin and Twist1, decreased the binding ability to the E-cadherin promoter. HMGA1 rescued the decrease of MMP2, p-Stat3 and EMT related genes expression in KIFC1 silenced HCC cells. Through modulating HMGA1 transcriptional activity, KIFC1 activated Jak/Stat pathway and promoted EMT in HCC cells thus accelerated HCC pathogenesis. Meanwhile, KIFC1 was upregulated by TCF-4, and KIFC1 inhibition in combination with paclitaxel displayed a synergistic effect to increase HCC drug sensitivity. Our observations provide new insights into understanding the underlying mechanisms of KIFC1 for HCC pathogenesis and reveal that KIFC1 could serve as an attractive anticancer agent against HCC, which deserves further exploration.

## Conclusions

To sum up, our results indicate that KIFC1 is a TCF-4 activated gene which is essential for the proliferation and metastasis of hepatocellular carcinoma. Mechanistically, KIFC1 promotes EMT by upregulating HMGA1 transcriptional activity. Besides, KIFC1 inhibition enhances HCC cell sensitivity to paclitaxel. Therefore, KIFC1 might be a promising HCC biomarker and potential target to suppress HCC progression.

## Additional files

Additional file 1: **Table S1.** Sequences of primers and shRNA used in this study. **Table S2.** The antibodies used in WB assay. (DOCX 21 kb)

Additional file 2: MS1-proteins-HepG2-vector. MS2-proteins-HepG2-KIFC1. MS3-HepG2-KIFC1 vs Vector (ZIP 72 kb)

Additional file 3: **Figure S1.** ERK, p-ERK, STAT3 and p-STAT3 were analyzed in KIFC1 knockdown and overexpression cells by western blotting. (TIF 1491 kb)

## Data Availability

All data generated or analyzed during this study are included in this published article and its supplementary information files.

## Abbreviations

DFS: disease-free survival
EMT: epithelial-mesenchymal transition
HCC: hepatocellular carcinoma
HMGA1: high mobility group AT-hook 1
KIFC1: kinesin family member C1
OS: overall survival
