# Correction to: KIFC1 is activated by TCF-4 and promotes hepatocellular carcinoma pathogenesis by regulating HMGA1 transcriptional activity

**DOI:** 10.1186/s13046-019-1460-0

**Published:** 2019-10-31

**Authors:** Kai Teng, Shi Wei, Chi Zhang, Jiewei Chen, Jinbin Chen, Kanghua Xiao, Jun Liu, Miaomiao Dai, Xinyuan Guan, Jingping Yun, Dan Xie

**Affiliations:** 1 State Key Laboratory of Oncology in South China, Collaborative Innovation Center for Cancer Medicine, Sun Yat-sen University Cancer Center, Guangzhou, 510060 China; 2 Department of Pathology, Sun Yat-sen University Cancer Center, Guangzhou, 510060 China; 3 Department of Hepatobiliary Oncology, Sun Yat-sen University Cancer Center, Guangzhou, 510060 China; 4 Department of Urology, Sun Yat-sen University Cancer Center, Guangzhou, 510060 China

**Correction to: J Exp Clin Cancer Res (2019) 38: 329**

**https://doi.org/10.1186/s13046-019-1331-8**

In the original publication of this article [1], the indicated stages (I-II III-IV) were missing in the clinical stage part at both Table 1 and Table 2. The revised tables are shown below.

**Table 1:** Correlation expression of KIFC1 and clinicopathological parameters in 168 HCC cases in SYSUCC cohort

Variable	All cases (*N* = 168)	KIFC1 expression (%)		*P* values
		Low expression (*N* = 97)	High expression (*N* = 71)	
Age (years)				0.763
≤ 51	90	51 (56.7)	39 (43.3)	
> 51	78	46 (59.0)	32 (41.0)	
Sex				0.849
Male	143	83 (58.0)	60 (42.0)	
Female	25	14 (56.0)	11 (44.0)	
AFP (ng/ml)				0.652
≤ 20	72	43 (59.7)	29 (40.3)	
> 20	96	54 (56.3)	42 (43.7)	
HBsAg				0.394
negative	11	5 (45.5)	6 (54.5)	
positive	157	92 (58.6)	65 (41.4)	
Cirrhosis				0.713
No	99	56 (56.6)	43 (43.4)	
Yes	69	41 (59.4)	28 (40.6)	
Necrosis				0.506
No	76	46 (60.5)	30 (39.5)	
Yes	92	51 (55.4)	41 (44.6)	
Clinical stage				0.009
I-II	111	72 (64.9)	39 (35.1)	
III-IV	57	25 (43.9)	32 (56.1)	
Histologic grade (WHO)				0.113
G1-G2	83	53 (63.9)	30 (36.1)	
G3-G4^*^	85	44 (51.8)	41 (48.2)	
Tumor size (cm)				0.031
≤ 5	73	49 (67.1)	24 (32.9)	
> 5	95	48 (50.5)	47 (49.5)	
Tumor multiplicity				0.688
Unifocal	128	75 (58.6)	53 (41.4)	
Multifocal	40	22 (55.0)	18 (45.0)	
Intravascular emboli				0.688
No	100	59 (59.0)	41 (41.0)	
Yes	68	38 (55.9)	30 (44.1)	
Recurrence				0.037
No	125	78 (62.4)	47 (37.6)	
Yes	43	19 (44.2)	24 (55.8)	

^*^:including 3 cases sarcomatoid liver cancer

**Table 2:** Univariate and multivariate analysis of different prognostic parameters in 168 HCC patients

Variable	Univariate analysis			Multivariate analysis	
	All cases	Mean survival (months)	*p* Value	HR (95% CI)	*p* Value
Age (years)			0.339		
≤ 51	90	49.799			
> 51	78	69.089			
Sex			0.061		
Male	143	69.106			
Female	25	43.696			
AFP (ng/ml)			0.055		
≤ 20	72	72.295			
> 20	96	53.431			
HBsAg			0.329		
negative	11	59.111			
positive	157	66.402			
Cirrhosis			0.829		
No	99	57.617			
Yes	69	66.272			
Necrosis			0.179		
No	76	60.350			
Yes	92	63.514			
Clinical stage			0.000		0.000
I-II	111	65.813		1	
III-IV	57	43.778		5.173 (2.456–10.894)	
Histologic grade (WHO)			0.000		0.035
G1-G2	82	65.254		1	
G3-G4^*^	86	55.882		2.334 (1.063–5.126)	
Tumor size (cm)			0.017		0.915
≤ 5	73	74.428			
> 5	95	49.049			
Tumor multiplicity			0.000		0.478
Unifocal	128	61.089			
Multifocal	40	50.483			
Intravascular emboli			0.013		0.483
No	100	61.257			
Yes	68	59.172			
Recurrence			0.007		0.061
No	125	70.974			
Yes	43	44.667			
KIFC1			0.002		0.012
Low		74.153		1	
High		45.568		2.371 (1.207–4.660)	

^*^:including 3 cases sarcomatoid liver cancer

The authors sincerely apologize for the inconvenience caused to the readers.
