# Correction to: KIFC1 is activated by TCF-4 and promotes hepatocellular carcinoma pathogenesis by regulating HMGA1 transcriptional activity

**DOI:** 10.1186/s13046-021-01913-x

**Published:** 2021-04-16

**Authors:** Kai Teng, Shi Wei, Chi Zhang, Jiewei Chen, Jinbin Chen, Kanghua Xiao, Jun Liu, Miaomiao Dai, Xinyuan Guan, Jingping Yun, Dan Xie

**Affiliations:** 1 State Key Laboratory of Oncology in South China, Collaborative Innovation Center for Cancer Medicine, Sun Yat-sen University Cancer Center, Guangzhou, 510060 China; 2 Department of Pathology, Sun Yat-sen University Cancer Center, Guangzhou, 510060 China; 3 Department of Hepatobiliary Oncology, Sun Yat-sen University Cancer Center, Guangzhou, 510060 China; 4 Department of Urology, Sun Yat-sen University Cancer Center, Guangzhou, 510060 China

**Correction to: J Exp Clin Cancer Res 38, 329 (2019)**

**https://doi.org/10.1186/s13046-019-1331-8**

Following publication of the original article [1], the authors identified minor errors in image-typesetting in Fig. 2 and Fig. 6. The specific panels that have been corrected are as follows:

Fig. 2e: HepG2-KIFC1-OE vector group (*lower left*)

Fig. 6b: 7402 shKIFC1+HMGA1 group (*lower right*)

The corrected figures are provided below. The corrections do not have any effect on the results or conclusions of the paper. The original article has been corrected.

**Fig. 2:**
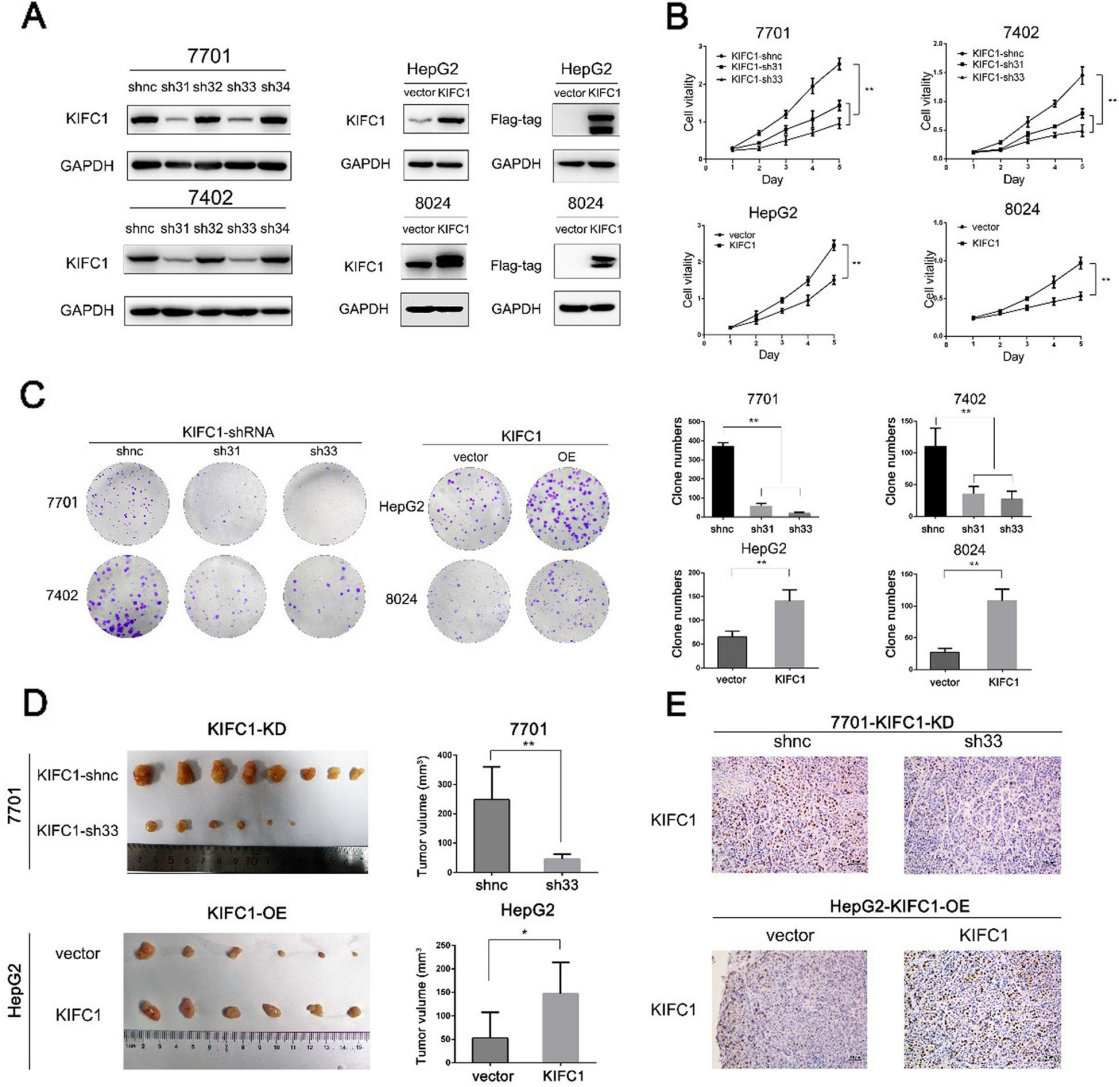
KIFC1 supports HCC cell proliferation in vitro and tumorigenicity in vivo.**a** Western blotting revealed that KIFC1 was efficiently knocked down in shRNA 31 (sh31) and shRNA 33 (sh33) and overexpressed in the corresponding cells. **b** The cell proliferation ability of the indicated cells was demonstrated by the CCK-8 assay. **c** Clone formation ability was tested in HCC cells with KIFC1 knockdown or overexpression. The clone numbers were counted and are presented in the right panels. Data are presented as the mean ± SD, * *P* < 0.01, ** *P* < 0.001. **d** Harvested xenografts formed by the KIFC1 knockdown 7701 and KIFC1 overexpression HepG2. Tumor volume was calculated and is presented in the right panels. Data are presented as the mean ± SD, * *P* < 0.01, ** *P* < 0.001. e Tissues from xenograft neoplasms by KIFC1 knockdown 7701 and KIFC1 overexpression HepG2 were tested by IHC for KIFC1 expression

**Fig. 6:**
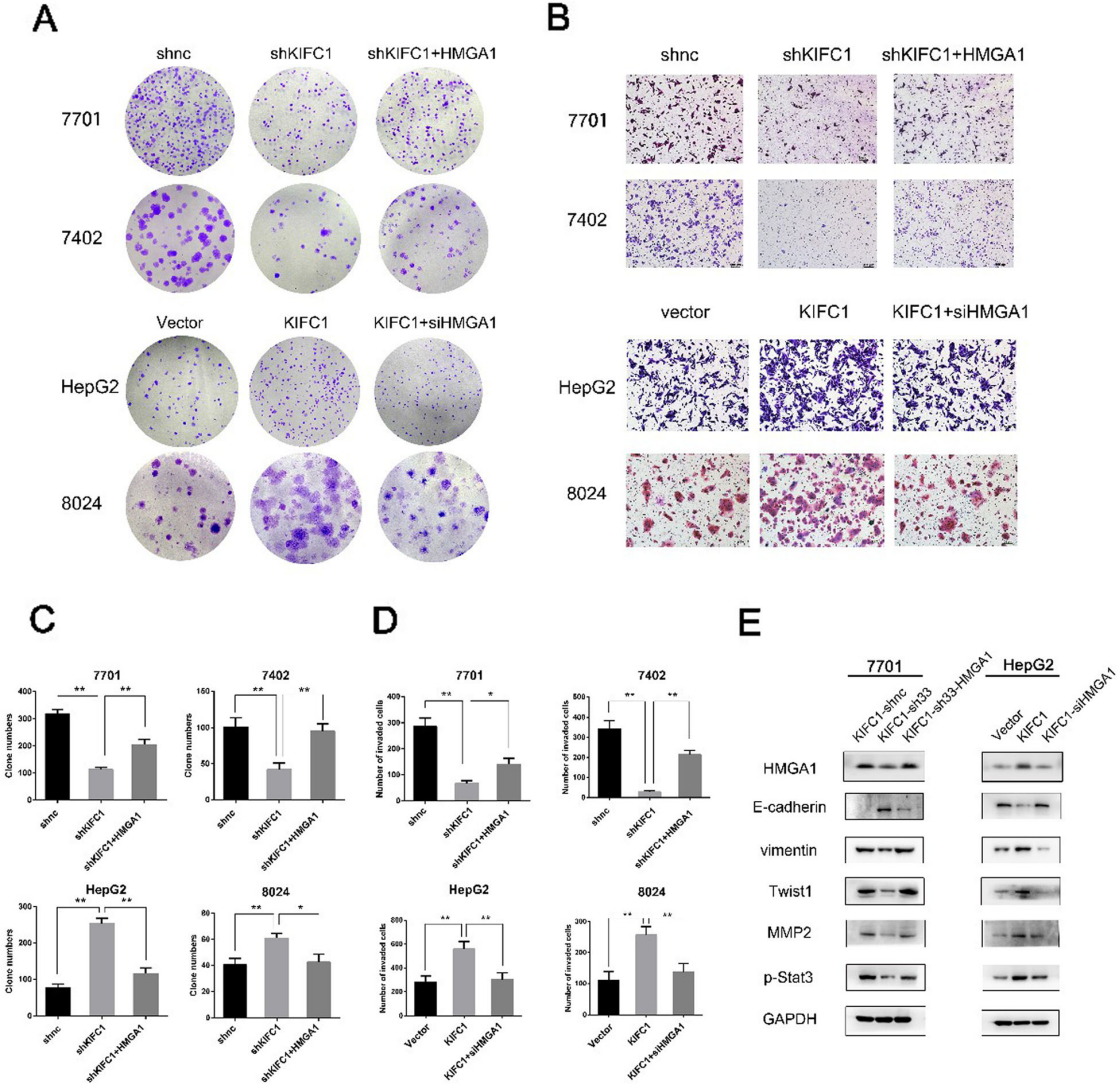
HMGA1 is responsible for KIFC1-enhanced HCC proliferation and invasion. **a** and **c** HMGA1 restored clone formation inhibited by KIFC1 knockdown. The counts of clone cell numbers are shown in panel **b**. shKIFC1 was used the same sequences as sh33 (5′- ccagggctatcaaataaagaa-3′). Data are presented as the mean ± SD, * *P* < 0.01, ** *P* < 0.001. **b** and **d** HMGA1 restored the invasive ability inhibited by KIFC1 knockdown. The counts of invaded cell numbers are shown in panel D. Data are presented as the mean ± SD, * *P* < 0.01, ** *P* < 0.001. **e** Western blot assay indicated that the levels of mesenchymal markers (vimentin, Twist1) as well as MMP2, p-Stat3 decreased while the level of epithelial marker E-cadherin increased in KIFC1 knockdown 7701 cells. This phenomenon could be reversed by enforced expression of HMGA1 in 7701 KIFC1 knockdown cells
