# Supplementary material for: KIFC1 is activated by TCF-4 and promotes hepatocellular carcinoma pathogenesis by regulating HMGA1 transcriptional activity
Source: J Exp Clin Cancer Res. 2019 Jul 24;38:329. doi: 10.1186/s13046-019-1331-8 (PMC6657086; doi:10.1186/s13046-019-1331-8)
Supplement: Supplementary file 1 — Table S1. Sequences of primers and shRNA used in this study. Table S2. The antibodies used in WB assay. (DOCX 21 kb) [file 13046_2019_1331_MOESM1_ESM.docx]

**Table S1 Sequences of primers and shRNA used in this study**

| **Name** | **Primer Sequence (5’-3’)** |
| --- | --- |
| Primers for qPCR | |
| GAPDH-F | TGCACCACCAACTGCTTAGC |
| GAPDH-R | GGCATGGACTGTGGTCATGAG |
| KIFC1-F | TGAGCAACAAGGAGTCCCAC |
| KIFC1-R | TCACTTCCTGTTGGCCTGAG |
| HMGA1-F | AGCGAAGTGCCAACACCTAAG |
| HMGA1-R | TGGTGGTTTTCCGGGTCTTG |
| ShRNA sequences | |
| KIFC1-ShNC | GCTTCGCGCCGTAGTCTTA |
| KIFC1-Sh31 | CCAGGGCTATCAAATAAAGAA |
| KIFC1-Sh33 | AAGCTACGTAGAGATCTACAAT |
| Primers for Duo luciferase reporter assay | |
| KIFC1-(-1000)-F | TGGGGGGATGGCAACATGGTT |
| KIFC1-(-500)-F | GACTCCAAAGGCCCTGAGCCC |
| KIFC1-(-300)-F | TAAATTTGGTGAGAAATGGCA |
| KIFC1-(universal)-R | ACGAACGCAACTGGCCAAGCG |
| KIFC1-(Mutation)-F | TGGGGGGATGGCAACATGGTT |
| KIFC1-(Mutation)-R | TTGAAGCCTGACAGTGGGACGATTTTTATCAGGAAAGAACTTTTCTTTATAGCCCTC |
| Primers for CHIP-PCR | |
| E-cadherin-F | GCCTGGCCCTATTGTTAC |
| E-cadherin-R | CCCAAAGCCTCAGTTTCTC |
| stat3-F | GCTTCTGTGCCCAAGTCC |
| stat3-R | AGAGGTCCCAGTGCGTGT |
| Vimentin-F | AAGAAGTCAGAATACGCTC |
| Vimentin-R | TGGTGTACTGAAAGGATTAA |
| Twist1-F | CCCTCTGGCTCTGCTGCCTTTC |
| Twist1-R | ATTTGCGTCCCGGCCTGCT |
| MMP2-F | ACCTGGGACCTTCCACTG |
| MMP2-R | TGGCTAGGACACCCTGCA |

**Table S2 The antibodies used in WB assay**

| **Antibody** | **Source** | **Catalogue number** | **Dilution** | **Company** |
| --- | --- | --- | --- | --- |
| **Primary antibodies for WB** | | | | |
| KIFC1 | Rabbit | [ab172620](https://www.abcam.com/kifc1-antibody-11445-ab172620.html) | 1:2000 | abcam |
| E-cadherin | Mouse | 610182 | 1:500 | BD |
| N-cadherin | Mouse | 610921 | 1:500 | BD |
| Vimentin | Mouse | 550513 | 1:1000 | BD |
| TWIST1 | Rabbit | T6451 | 1:500 | Sigma |
| MMP2 | Mouse | 66366 | 1:500 | Proteintech |
| Stat3 | Rabbit | 30835 | 1:1000 | Cell signaling technology |
| p-Stat3 | Rabbit | 9145 | 1:1000 | Cell signaling technology |
| ERK | Rabbit | 4695 | 1:1000 | Cell signaling technology |
| p-ERK | Rabbit | 4370 | 1:1000 | Cell signaling technology |
| β-catenin | Rabbit | 8480 | 1:1000 | Cell signaling technology |
| Gsk-3β | Rabbit | 12456 | 1:1000 | Cell signaling technology |
| p-Gsk-3β | Rabbit | 5558 | 1:1000 | Cell signaling technology |
| Axin2 | Rabbit | 2151 | 1:1000 | Cell signaling technology |
| Cyclin D1 | Rabbit | 2978 | 1:1000 | Cell signaling technology |
| C-myc | Rabbit | 5605 | 1:1000 | Cell signaling technology |
| Flag-tag | Rabbit | 14793s | 1:1000 | Cell signaling technology |
| Myc-tag | Mouse | M192-3B | 1:1000 | MBL |
| HMGA1 | Rabbit | ab129153 | 1:1000 | abcam |
| **Secondary antibodies for WB** | | | | |
| GAPDH | Mouse | 60004 | 1:5000 | Proteintech |
