# Supplementary figures and images for: KIFC1 is activated by TCF-4 and promotes hepatocellular carcinoma pathogenesis by regulating HMGA1 transcriptional activity
Source: J Exp Clin Cancer Res. 2019 Jul 24;38:329. doi: 10.1186/s13046-019-1331-8 (PMC6657086; doi:10.1186/s13046-019-1331-8)

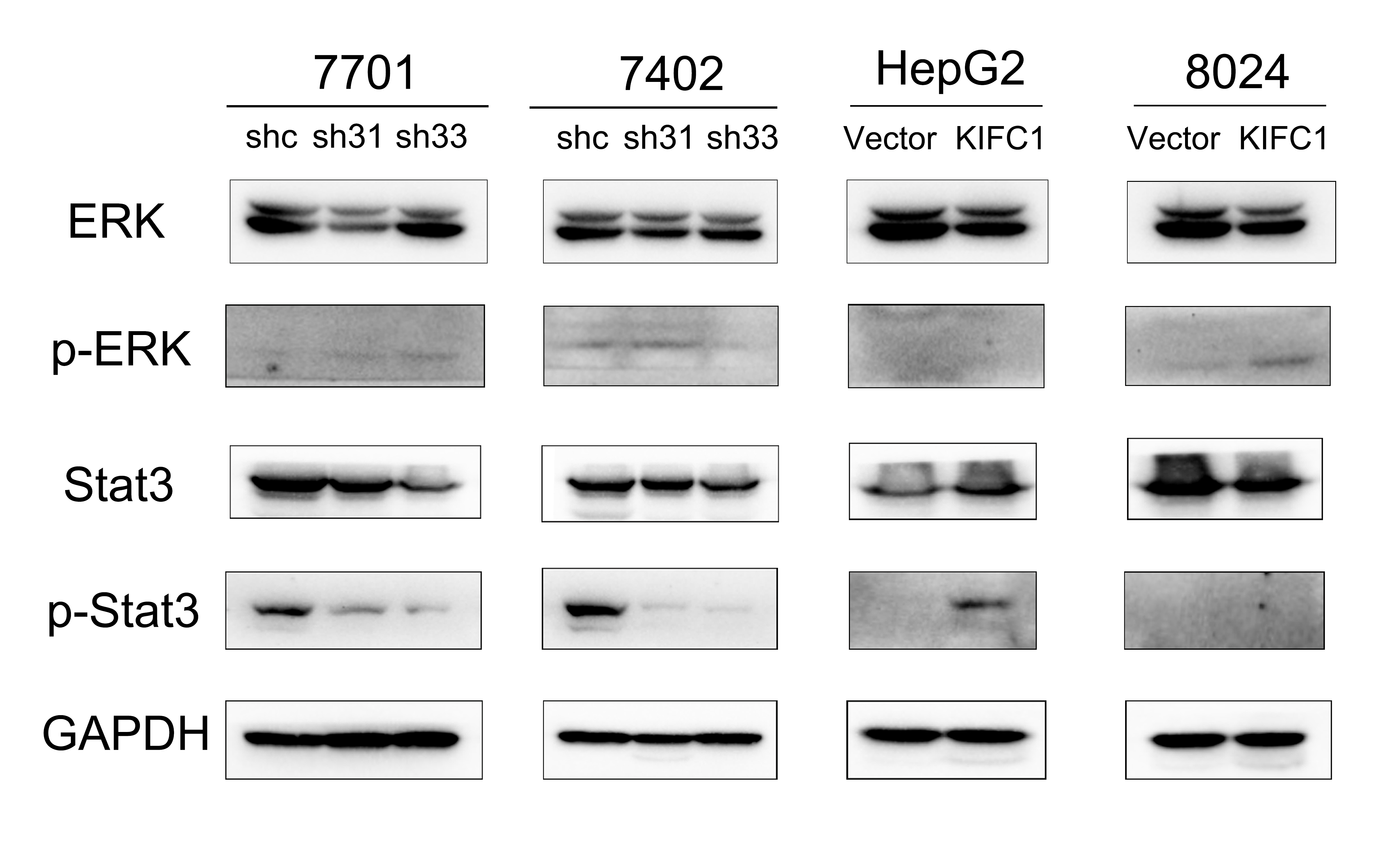

Supplement: Supplementary file 3 — Figure S1. ERK, p-ERK, STAT3 and p-STAT3 were analyzed in KIFC1 knockdown and overexpression cells by western blotting. (TIF 1491 kb) [file 13046_2019_1331_MOESM3_ESM.tif]
